# CENP-B creates alternative epigenetic chromatin states permissive for CENP-A or heterochromatin assembly

**DOI:** 10.1242/jcs.243303

**Published:** 2020-08-11

**Authors:** Koichiro Otake, Jun-ichirou Ohzeki, Nobuaki Shono, Kazuto Kugou, Koei Okazaki, Takahiro Nagase, Hisashi Yamakawa, Natalay Kouprina, Vladimir Larionov, Hiroshi Kimura, William C. Earnshaw, Hiroshi Masumoto

**Affiliations:** 1 Laboratory of Chromosome Engineering, Department of Frontier Research and Development, Kazusa DNA Research Institute, 2-6-7 Kazusa-Kamatari, Kisarazu 292-0818, Japan; 2 Public Relations and Research Promotion Group, Kazusa DNA Research Institute, 2-6-7 Kazusa-Kamatari, Kisarazu 292-0818, Japan; 3 Clinical Analysis Team, Department of Omics Research and Development, Kazusa DNA Research Institute, 2-6-7 Kazusa-Kamatari, Kisarazu 292-0818, Japan; 4 Genome Structure and Function Group, Developmental Therapeutics Branch, Center for Cancer Research, National Cancer Institute, National Institutes of Health, Bethesda, MD 20892, USA; 5 Cell Biology Unit, Institute of Innovative Research, Tokyo Institute of Technology, Yokohama 226-8503, Japan; 6 Wellcome Trust Centre for Cell Biology, University of Edinburgh, Edinburgh EH9 3BF, UK

**Keywords:** Centromere, CENP-B, ASH1L, HP1, Alternative epigenetic states, Acidic-rich domain

## Abstract

CENP-B binds to CENP-B boxes on centromeric satellite DNAs (known as alphoid DNA in humans). CENP-B maintains kinetochore function through interactions with CENP-A nucleosomes and CENP-C. CENP-B binding to transfected alphoid DNA can induce *de novo* CENP-A assembly, functional centromere and kinetochore formation, and subsequent human artificial chromosome (HAC) formation. Furthermore, CENP-B also facilitates H3K9 (histone H3 lysine 9) trimethylation on alphoid DNA, mediated by Suv39h1, at ectopic alphoid DNA integration sites. Excessive heterochromatin invasion into centromere chromatin suppresses CENP-A assembly. It is unclear how CENP-B controls such different chromatin states. Here, we show that the CENP-B acidic domain recruits histone chaperones and many chromatin modifiers, including the H3K36 methylase ASH1L, as well as the heterochromatin components Suv39h1 and HP1 (HP1α, β and γ, also known as CBX5, CBX1 and CBX3, respectively). ASH1L facilitates the formation of open chromatin competent for CENP-A assembly on alphoid DNA. These results indicate that CENP-B is a nexus for histone modifiers that alternatively promote or suppress CENP-A assembly by mutually exclusive mechanisms. Besides the DNA-binding domain, the CENP-B acidic domain also facilitates CENP-A assembly *de novo* on transfected alphoid DNA. CENP-B therefore balances CENP-A assembly and heterochromatin formation on satellite DNA.

## Abstract (precis)

**Summary:** The acidic domain of CENP-B facilitates assembly of various proteins at centromeric satellite DNA, including HP1 and ASH1L. CENP-B controls centromere epigenetic status.

## INTRODUCTION

The centromere is a specialized genetic locus that plays an essential role in chromosome segregation. Centromeric nucleosomes contain the specific histone H3 variant CENP-A (Earnshaw and Rothfield, 1985; Palmer et al., 1987; Black and Cleveland, 2011), an epigenetic mark that directs the assembly of other centromere proteins (for example, the constitutive centromere-associated network, CCAN, or the interphase centromere complex, ICEN) and outer kinetochore components (Foltz et al., 2006; Izuta et al., 2006; Okada et al., 2006; Allshire and Karpen, 2008; Cheeseman and Desai, 2008; Fukagawa and Earnshaw, 2014; Westhorpe and Straight, 2015; Musacchio and Desai, 2017). CENP-A nucleosomes are stabilized by interactions with other CCAN components, including CENP-C (Carroll et al., 2010; Fachinetti et al., 2013) and CENP-N (Guo et al., 2017).

The modification status of centromeric histones is important for CENP-A assembly (Bergmann et al., 2011; Shang et al., 2016). Methylation of histone H3 lysine 4 (H3K4me) and lysine 36 (H3K36me) is observed around CENP-A chromatin (Sullivan and Karpen, 2004; Bergmann et al., 2011; Bailey et al., 2016). Dimethylation of H3K4 (H3K4me2) is required for the recruitment of HJURP (Bergmann et al., 2011) and for preventing flanking heterochromatin from spreading into the centromere (Molina et al., 2016).

At human centromeres, CENP-A assembles on a portion of a large repetitive DNA locus containing α-satellite DNA (alphoid DNA) (Willard and Waye, 1987). Most of the flanking satellite DNA regions (pericentromeres) are occupied by heterochromatin (Sullivan and Karpen, 2004) enriched with Suv39h1-mediated trimethylation of histone H3 lysine 9 (H3K9me3), which recruits HP1 (HP1α, β and γ, also known as CBX5, CBX1 and CBX3, respectively). Heterochromatin is reported to have important roles in chromosome stability and maintenance of sister chromatid cohesion during chromosome segregation (Peters et al., 2001; Grewal and Jia, 2007). Thus, both centromere chromatin and heterochromatin are important for accurate chromosome segregation processes.

CENP-B (Earnshaw et al., 1987a), the only known centromeric DNA sequence-specific binding protein in metazoans, binds to a 17-bp CENP-B box sequence in human alphoid DNA or mouse minor satellite DNA (Masumoto et al., 1989) via its N-terminal DNA-binding domain (DBD) (Pluta et al., 1992; Muro et al., 1992).

CENP-B is required for *de novo* CENP-A assembly to generate human artificial chromosomes (HACs) (Harrington et al., 1997; Ikeno et al., 1998) that segregate during cell division similarly to natural chromosomes (Tsuduki et al., 2006). When the CENP-B boxes on input alphoid DNA are mutated, HAC formation is diminished (Ohzeki et al., 2002). *De novo* CENP-A assembly also fails when alphoid DNA is introduced into CENP-B knockout (KO) mouse embryonic fibroblast (MEF) cells (Okada et al., 2007). In contrast, CENP-B is not required for the maintenance of established centromeres. No CENP-B box is found on the Y chromosome alphoid DNA (Masumoto et al., 1989; Earnshaw et al., 1991) or ectopically formed neocentromeres on chromosome arms (du Sart et al., 1997; Alonso et al., 2003). In addition, CENP-B KO mice are viable and fertile for at least one generation (Kapoor et al., 1998; Hudson et al., 1998; Perez-Castro et al., 1998).

Recent studies suggest a possible explanation for why CENP-B might not be essential for centromere maintenance and function. CENP-B helps stabilize kinetochore structure through interaction with CENP-A nucleosomes and CENP-C via its N- and C-terminal domains, respectively (Suzuki et al., 2004; Fachinetti et al., 2015; Fujita et al., 2015). However, CENP-C also binds to the C-terminal tail of nucleosomal CENP-A (Carroll et al., 2010; Fachinetti et al., 2013). Thus, the CENP-C–CENP-A direct interaction can bypass the requirement for CENP-B. In the absence of CENP-B, mutation of the C-terminal tail of CENP-A perturbs chromosome segregation because of the loss of the connection between CENP-C and centromeric chromatin (Fachinetti et al., 2015; Hoffmann et al., 2016). Therefore, CENP-B has important roles not only in *de novo* centromere formation, but also in centromere maintenance as a backup system.

In contrast to its function in CENP-A assembly and stabilization, CENP-B can also promote heterochromatinization. In CENP-B KO MEF cells containing human alphoid DNA integrated on a chromosome arm, CENP-B expression results in increased deposition of H3K9me3 on the alphoid DNA by Suv39h1 (Okada et al., 2007). Our previous studies using a synthetic alphoid DNA sequence containing a tetR binding site, tetO (alphoid^tetO^), in the alphoid^tetO^ HAC revealed that tethering of heterochromatin factors to the alphoid^tetO^ HAC inhibits CENP-A assembly (Nakano et al., 2008; Cardinale et al., 2009; Ohzeki et al., 2012; Shono et al., 2015; Martins et al., 2016). Thus, CENP-B can promote alternative epigenetic chromatin states favoring either CENP-A assembly or heterochromatin formation on the same alphoid DNA. The underlying mechanism for this ‘bidirectional’ behavior is unknown.

In this study, we analyzed the function of CENP-B and identified proteins assembled by each CENP-B sub-domain. We identified not only the heterochromatin-promoting methyltransferase Suv39h1 and its binding protein HP1, but also new open chromatin-related modifiers and centromere proteins. Interestingly, one of the identified factors, H3 lysine 36 methyltransferase ASH1L, facilitated open chromatin formation competent for *de novo* CENP-A assembly on exogenous alphoid DNA in HT1080 cells. In addition, ASH1L depletion increased heterochromatin formation, but decreased CENP-A assembly on endogenous centromeric alphoid DNA in HeLa cells. ASH1L might be a key factor rendering CENP-B-bound alphoid chromatin competent for CENP-A assembly by avoiding excessive heterochromatinization.

## RESULTS

### Exploration of factors that assemble on alphoid chromatin in a CENP-B-dependent manner

**Fig. 1.**
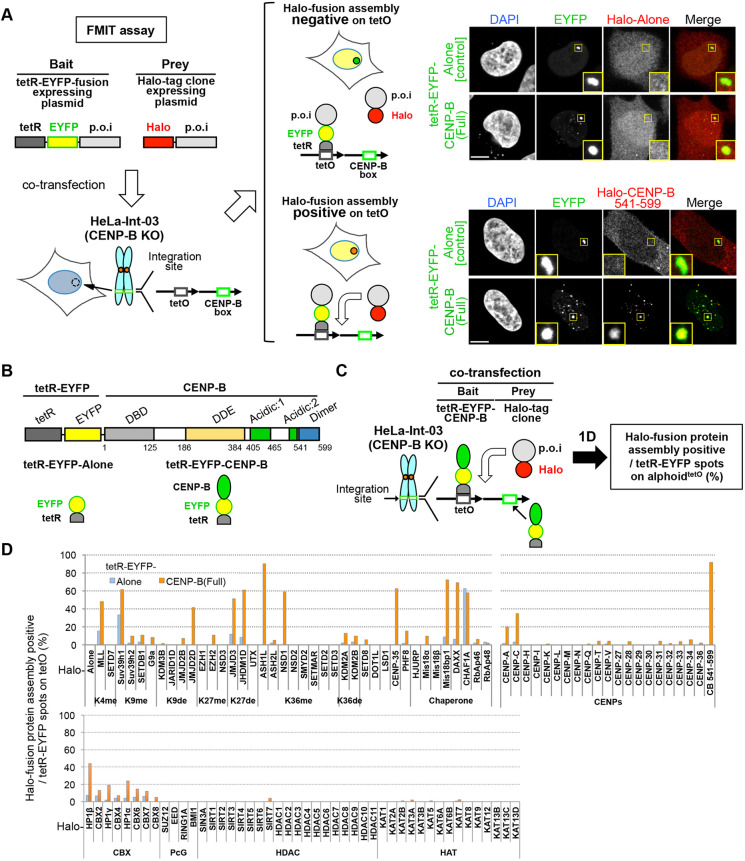
**Screening of proteins for CENP-B-dependent assembly on ectopic alphoid^tetO^ DNA using a fluorescence microscopy-based interaction-trap (FMIT) assay.** (A) Experimental design of the FMIT assay. Expression plasmids of a bait or prey are co-transfected into the CENP-B KO HeLa-Int-03 cell line, which has a synthetic alphoid repeat array containing tetO and CENP-B box sequences (alphoid^tetO^ DNA) at an ectopic integration site. Colocalization of EYFP signal and the Halo tag-TMR ligand signal can be detected when an interaction exists between bait and prey and/or when the prey recognizes an alphoid^tetO^ chromatin change induced by the bait. Negative and positive example images are shown in the upper right and lower right panels, respectively. In the positive case, tetR–EYFP–CENP-B^Full^ recruits the Halo-tag fused to the CENP-B dimer domain (Halo–CENP-B 541–599). The yellow squares indicate the location of tetR–EYFP protein spots on the alphoid^tetO^ DNA site (shown magnified in inset images). Scale bars: 5 µm. (B) Schematic structure of the bait (tetR–EYFP–CENP-B) and negative control (tetR–EYFP alone) used in this figure. DBD, DNA-binding domain; DDE, DDE-superfamily endonuclease domain; Acidic:1, acidic domain 1; Acidic:2, acidic domain 2; Dimer, dimerization domain. (C) Exploration of protein assembly on the ectopic alphoid^tetO^ DNA integration site by CENP-B tethering. The bait and prey expression plasmids were co-transfected into the HeLa-Int-03 CENP-B KO cell line. The cells were fixed 1 d after transfection. More than 50 cells were analyzed to calculate the frequency of Halo positive EYFP-spots for each prey. The tetR–EYFP–CENP-B binds to not only tetO, but also to the CENP-B box. (D) CENP-B-independent (tetR–EYFP alone bait, blue bars) and CENP-B-dependent (tetR–EYFP–CENP-B bait, orange bars) assembly of Halo-tag fused histone modifiers, chaperones, CENPs and heterochromatin proteins was evaluated as the percentage of cells exhibiting colocalization of bait and prey on the ectopic alphoid^tetO^ integration site. K4me, K9me, K27me and K36me indicate H3 lysine methyl transferases. K9de, K27de and K36de indicate H3 lysine demethylases. CBX, chromobox proteins; PcG, polycomb-group proteins; HDAC, histone deacetylases; HAT, histone acetyltransferases.

CENP-B-dependent factors that assemble on alphoid DNA might be involved in bidirectional chromatin formation. We used a fluorescence microscopy-based interaction-trap (FMIT) assay (Shono et al., 2015; Ohzeki et al., 2016) to identify centromere proteins and histone modifiers recruited to ectopic alphoid^tetO^ DNA by tetR–EYFP–CENP-B in the HeLa-Int-03 CENP-B KO cell line. This cell line was obtained by knocking out the CENP-B gene of HeLa-Int-03 to eliminate the influence of endogenous CENP-B on the ‘prey’ assembly (Ohzeki et al., 2012) (Fig. S1A–D). This assay detects the recruitment of proteins of interest (POI) at the ectopic chromosomal site within 1–2 days after tetR–EYFP–CENP-B transfection (Fig. 1A).

First, we screened for factors that assemble at the ectopic alphoid^tetO^ DNA integration site. Prior to transfection, this array lacks detectable CENP-A and is covered with heterochromatin (Ohzeki et al., 2012). We used tetR–EYFP-CENP–B^full-length^ as bait (Fig. 1B) and a library of Halo-fused proteins as prey. This analysis (Fig. 1C,D) confirmed, as previously reported, that CENP-B recruits both the centromere proteins, CENP-A, CENP-C and CENP-B^541–599^ (a control dimerization domain), as well as the heterochromatin-promoting factor Suv39h1 to the ectopic alphoid^tetO^ DNA array (Fig. S1E) (Kitagawa et al., 1995; Suzuki et al., 2004; Okada et al., 2007; Fujita et al., 2015). In addition to the aforementioned proteins, CENP-B also recruited various histone modifiers [ASH1L, NSD1, MLL (also known as KMT2A), JMJD2D (KDM4D), JMJD3 (KDM6B), JHDM1D (KDM7A) and CENP-35], histone chaperones (DAXX), histone chaperone recruitment factor (Mis18BP1) and heterochromatin proteins (HP1α, β and γ, referred to here collectively as HP1s) to the ectopic integration site. We considered these factors as candidates for the bidirectional alternative epigenetic regulation of chromatin.

### Evaluation of factors shown to promote endogenous CENP-A assembly on the HAC centromere

**Fig. 2.**
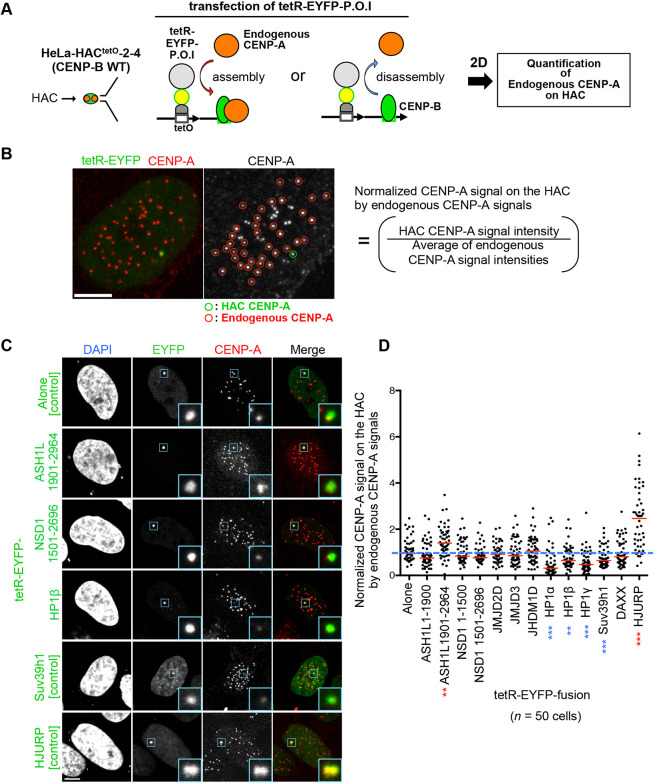
**Tethering of ASH1L increases CENP-A levels on HAC centromeres.** (A) Schematic showing quantification of CENP-A assembly on an alphoid^tetO^ HAC. A HeLa cell line containing an alphoid^tetO^ HAC (HeLa-HAC^tetO^-2-4) was transfected with tetR–EYFP-fusion expression plasmids and fixed 2 d after transfection for immunostaining with anti-CENP-A antibody. The red and blue arrows indicate the effect on endogenous CENP-A (assembly or disassembly) of tetR­–EYFP-fusion tethering. (B) Example of quantification of CENP-A assembly on the HAC. HeLa-HAC^tetO^-2-4 cells transfected with tetR–EYFP alone were stained with anti-CENP-A antibody. Scale bar: 5 µm. For quantification, CENP-A signal intensity on the HAC (green circle) is normalized by the average signal intensity of the endogenous centromeres (red circles) as described in Shono et al. (2015). (C,D) Quantification of CENP-A assembly on the HAC by tethering of the identified factors (right panel). Example images of the quantification assay are shown in the left panel (blue squares indicate the HAC, shown magnified in inset images). Scale bar: 5 µm. Fifty cells were analyzed for each assay. Blue dashed line indicates relative values to the median of control (Alone). Red lines indicate the median. **P<0.01, ***P<0.001 compared to control (Mann–Whitney test; red asterisk, increase; blue asterisk, decrease).

Next, we evaluated whether each candidate obtained in the experiments shown in Fig. 1 had a positive or negative impact on centromeric CENP-A levels. Previous studies showed that endogenous CENP-A levels on the alphoid^tetO^ HAC can be increased by tethering CENP-A deposition factors (e.g. HJURP) (Dunleavy et al., 2009; Foltz et al., 2009), CENP-A nucleosome stabilization factors and kinetochore proteins interacting with CENP-C to the HAC (Shono et al., 2015). Conversely, tethering of heterochromatin factors (e.g. Suv39h1) decreased CENP-A levels. We therefore evaluated whether the additional factors identified here affect CENP-A levels on the alphoid^tetO^ HAC (HeLa-HAC-2-4) (Fig. 2A,B).

We could not detect EYFP signals following transfection with constructs expressing full-length tetR–EYFP–ASH1L and tetR–EYFP–NSD1. These proteins were therefore sub-divided into regions including the C-terminal SET domain and the remaining N-terminal region. Besides tetR–EYFP–HJURP, tethering of tetR–EYFP–ASH1L^1901–2964^ also enhanced the endogenous CENP-A signal on the alphoid^tetO^ HAC (Fig. 2C,D). Conversely, tethering of the heterochromatin factors HP1α, HP1β, HP1γ and Suv39h1 decreased CENP-A, as previously reported (Shono et al., 2015; Martins et al., 2016). We therefore focused on ASH1L, which increases centromeric CENP-A levels, and HP1, which decreases centromeric CENP-A levels, in the following analyses.

### The acidic domain of CENP-B facilitates assembly of ASH1L, HP1 and many other factors

**Fig. 3.**
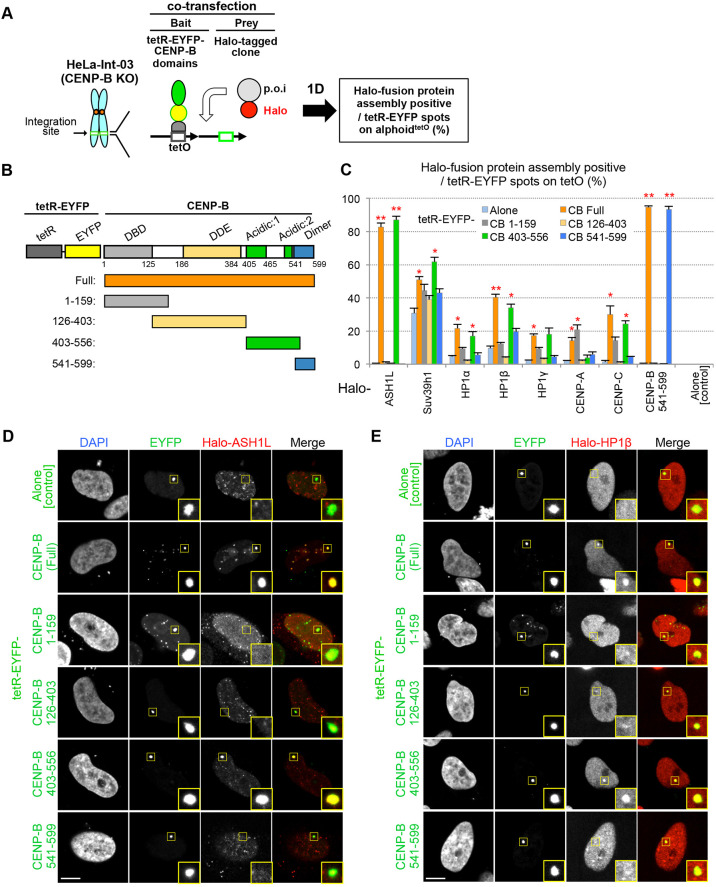
**Identification of CENP-B domains that assemble ASH1L and HP1, using the FMIT assay.** (A,B) Schematic drawing of FMIT assay (A) and tested tetR–EYFP–CENP-B deletion series bait (B). (C) Frequency of assembly of Halo-fused proteins on the ectopic alphoid^tetO^ DNA integration site. The tetR–EYFP–CENP-B series, tetR­–EYFP alone and Halo-tag clone (Halo-fused gene) expression plasmids were co-transfected into the HeLa Int-03 CENP-B KO cell line. More than 50 cells were counted to obtain the frequency of Halo-positive EYFP-spots for each assay. Results are mean±s.e.m. (*n*=3 experiments). *P*-values against tetR–EYFP alone (two-tailed *t*-test) are indicated by asterisks. *P<0.05, **P<0.01. (D,E) Representative images of Halo–ASH1L (D) and Halo–HP1β (E) assembly in the FMIT assay with the tetR–EYFP–CENP-B series bait. Cells were stained with DAPI and Halo-TMR ligand. The yellow squares indicate tetR–EYFP–protein spots on the alphoid^tetO^ DNA site, and are shown magnified in inset images. Scale bars: 5 µm.

We next performed FMIT assays using different domains of CENP-B (tetR–EYFP–CENP-B^domains^) as bait to investigate which region of CENP-B is involved in recruitment of each of the factors identified in the initial screen (Fig. 3A,B). Consistent with previous studies, Halo-tagged CENP-A, CENP-C and CENP-B^541–599^ prey were recruited by CENP-B^1–159^, CENP-B^403–556^ or CENP-B^541–599^ bait fusion proteins, respectively (Fig. 3C; Fig. S2A). Halo–ASH1L and Halo–HP1s were recruited most effectively by the CENP-B acidic domain (tetR–EYFP–CENP-B^403–556^) with similar efficiency to full-length CENP-B bait (tetR–EYFP–CENP-B^Full^) (Fig. 3C–E). Interestingly, a wide range of other factors were also recruited by the CENP-B acidic domain. These included CENP-A assembly factor Mis18BP1 (Fujita et al., 2007), histone H3.3 chaperone DAXX (Drané et al., 2010), heterochromatin-related factor Suv39h1, open chromatin-related factors H3K4 dimethylase MLL and H3K36 methyltransferase NSD1 and others (JMJD2D, JMJD3, JHDM1D, CENP-35) (data not shown). Immunostaining and chromatin immunoprecipitation (ChIP) analysis using specific antibodies confirmed that endogenous ASH1L and HP1β were indeed recruited to the ectopic site by tethered tetR–EYFP–CENP-B^Full^ and tetR–EYFP–CENP-B^403–556^ (Fig. S2B). Thus, the tethering did not require overexpression of the Halo-tagged bait proteins.

We next used the FMIT assay to identify which ASH1L domain is required for CENP-B-dependent localization to alphoid DNA, using tetR–EYFP–CENP-B^403–556^ as bait and a Halo–ASH1L deletion series as prey. The assembly frequencies of Halo–ASH1L truncations at the ectopic alphoid^tetO^ DNA integration site following tethering of CENP-B^403–556^ were ∼8% (ASH1L^1–824^), ∼77% (ASH1L^800–1900^), and ∼3% (ASH1L^1901–2964^). Thus, ASH1L^800–1900^, which contains the AT-hook domain, is apparently responsible for CENP-B^403–556^-dependent ASH1L recruitment (Fig. S2C). In the reverse bait and prey combination, recruitment of Halo–CENP-B^160–556^ was observed only following ASH1L^1–1900^ tethering, with weaker signals than the inverse combination (∼26%) (data not shown). Thus, ASH1L is recruited to alphoid DNA by the CENP-B acidic domain, apparently via the ASH1L^800–1900^ domain containing AT-hook-motifs.

### CENP-B facilitates ASH1L and HP1β localization to alphoid DNA

**Fig. 4.**
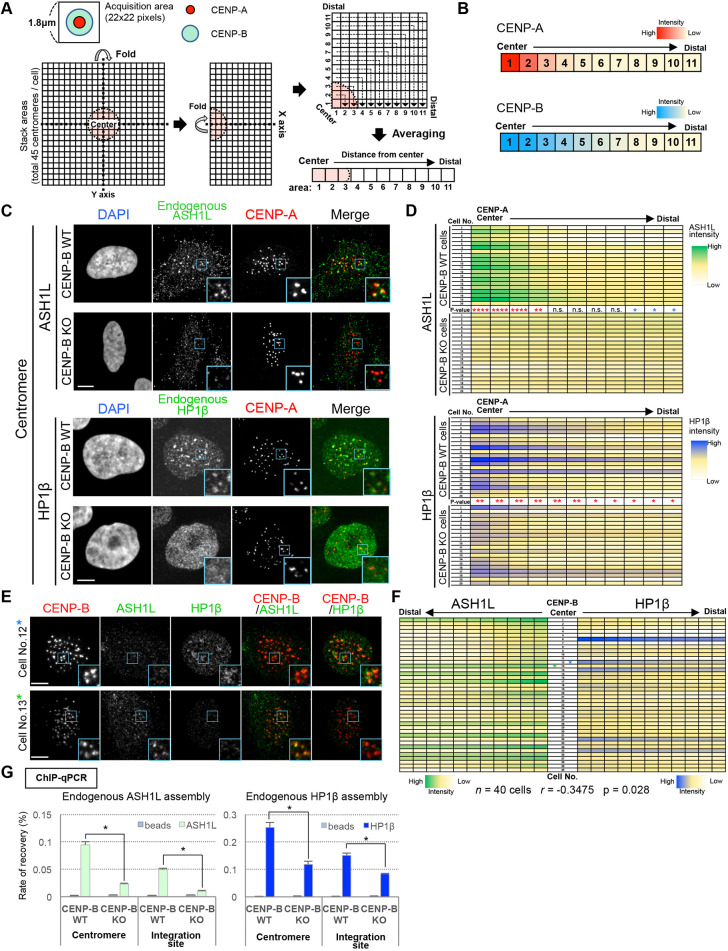
**CENP-B-dependent localization of ASH1L and HP1β to alphoid DNA.** (A) The fluorescence intensity distribution of proteins of interest (e.g. CENP-A and CENP-B) in a 22×22 pixel area centered on the fluorescence spot was quantified and integrated for each cell (see more details in Materials and Methods). (B) Example of the distribution of fluorescence intensity. CENP-A or CENP-B spots were analyzed for 20 cells, as described in A, each with 45 or 40 spots per cell. The quantitated intensities of the 20 cells were averaged for each distance and shown as heatmaps, with the intensity color scale indicated. (C,D) Detection of endogenous ASH1L or HP1β at centromeres. CENP-B^WT^ and CENP-B^KO^ HeLa-Int-03 cell lines were stained with DAPI, anti-CENP-A and anti-ASH1L or HP1β antibody. (C) Example images of the staining (anti-CENP-A antibody, red; anti-ASH1L or HP1β antibody, green). Blue square areas indicate examples of centromeres shown enlarged in inset images. Scale bars: 5 µm. (D) Heatmap of signal intensity profile of ASH1L and HP1β around CENP-A spots. The signals were quantified as described in A. Twenty cells were analyzed for each cell line (45 centromeres per cell). The signal intensity indicator is displayed to the right of the heat map. *P*-value indicates a significant difference in each area between WT and KO. ****P<0.0001; **P<0.001; *P<0.005; n.s., not significant (Mann–Whitney test; red, WT>KO; blue, WT<KO). (E,F) Cells were stained with anti-CENP-B (detected using an Alexa Fluor 405-conjugated secondary antibody), anti-ASH1L (Alexa Fluor 594) and anti-HP1β (Alexa Fluor488) antibody. (E) Example images of the staining are shown for cell 12 and cell 13 (indicated by asterisks in F). CENP-B, red; ASH1L, green; HP1β, green. Blue squares indicate example areas of centromeres shown enlarged as inset images. Scale bars: 5 µm. (F) Heatmap of signal intensity profile of ASH1L and HP1β around CENP-B spots. In total, 40 cells were analyzed (with 40 CENP-B spots per cell). The signals were quantified as described in A. The relationship between distributions of ASH1L and HP1β around CENP-B was measured by total signal intensities of the pixels where significant difference was detected in D (i.e. pixel area 1–4 for ASH1L and pixel area 1–11 for HP1β). Spearman rank correlation coefficient; r=−0.3475; *P*=0.028. (G) Quantification of endogenous ASH1L or HP1β assembly on centromeres (Alphoid^Chr.21^) and integration sites (Alphoid^tetO^) using ChIP. Results are mean±s.e.m. (*n*=3 experiments). *P<0.05 (two-tailed *t*-test).

ASH1L is a trithorax-group protein that methylates H3K36, and is reported to be mutually exclusive with polycomb group-mediated H3K27-methylated facultative heterochromatin (Schuettengruber et al., 2007; Miyazaki et al., 2013). ASH1L localization and function at centromeres has not previously been reported, but H3K36me2/3 has been reported to be present at centromeres (Bergmann et al., 2011; Bailey et al., 2016). Therefore, we first investigated whether endogenous ASH1L and HP1β localize to centromeres in a manner dependent on CENP-B binding [comparing wild-type CENP-B (CENP-B^WT^) and CENP-B^KO^ in HeLa-Int-03 cells] using anti-ASH1L or anti-HP1 antibodies combined with an anti-CENP-A antibody. HP1β is distributed across a rather wide area of the centromere, including the pericentromere, and does not localize as a focused spot like the CENP proteins and CCAN. Therefore, we developed the approach shown in Fig. 4A to map the distribution of ASH1L and HP1β fluorescence intensity at centromeres. We quantified and integrated the ASH1L and HP1β fluorescence intensity across a region from the pixel at the center of the CENP-A signal out to a radius of 11 pixels. We did this for each of the 45 centromeric CENP-A signals per cell. The distributions of fluorescence intensity of ASH1L and HP1β were both significantly higher in the CENP-A-proximal region in CENP-B^WT^ cells. However, this gradient of localization was lost in CENP-B^KO^ cells (Fig. 4B–D).

Next, we demonstrated by immunostaining and ChIP analysis that CENP-B binding to an ectopic alphoid^tetO^ DNA integration array also recruited endogenous ASH1L and HP1β in HeLa-Int-03 cells (array visualized by tethering tetR–EYFP, Fig. S3B,C). The ChIP recovery rate of ASH1L with alphoid DNA was reduced by 75% in the chromosome 21 centromere, and 80% in the integration site, in CENP-B^KO^ cells compared to those in CENP-B^WT^ cells (Fig. 4G). Similarly, the recovery rate of HP1β was reduced by 53% in the centromere and 47% in the integration site. In CENP-B^KO^ cells, no significant decrease was detected in the total amount of cellular ASH1L and HP1β (Fig. S3A). Thus, ASH1L and HP1β assembly on alphoid DNA requires CENP-B binding.

These analyses also indicated that ASH1L and HP1β are not equally distributed around the centromeres of each cell. When CENP-B, ASH1L and HP1β were triple-stained and analyzed by the above method (Fig. 4A), it was found that ASH1L fluorescence was weak in cells where HP1β fluorescence was strong in the centromere region. Conversely, HP1β fluorescence was weak in cells where ASH1L fluorescence was strong in the centromere region (Fig. 4E,F). We conclude that ASH1L and HP1β localization to centromeres appears to be mutually exclusive.

### ASH1L and HP1 assemblies exhibit mutually exclusive properties

We have shown that the CENP-B acidic region (403–556) recruits ASH1L and/or HP1 to an ectopic alphoid^tetO^ array (Fig. 3) and that CENP-B also recruits both ASH1L and/or HP1 to endogenous centromeres (Fig. 4). However, the effects of ASH1L and HP1 on CENP-A chromatin assembly are diametrically opposed (Fig. 2C,D). We therefore asked how the binding of ASH1L or HP1β influences each other and centromere function.

**Fig. 5.**
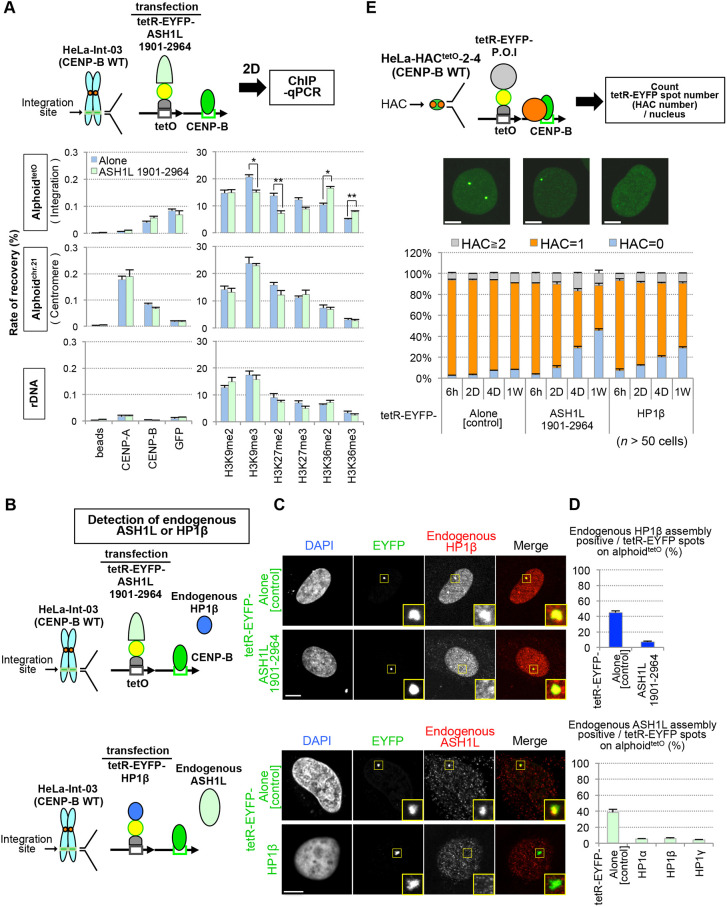
**Mutually exclusive assembly of ASH1L and HP1 on alphoid DNA.** (A) Histone modification changes promoted by ASH1L tethering. The tetR–EYFP alone or tetR–EYFP–ASH1L^1901–2964^ plasmid was transfected into HeLa-Int-03 cells. The cells were fixed at 2 d after transfection for ChIP analysis. Chromatin immunoprecipitated with antibodies against the proteins and modifications indicated were quantified by PCR. rDNA is shown as a control. Beads, without antibody for negative control. Results are mean±s.e.m. (*n*=3 experiments). *P<0.05; **P<0.01 (two-tailed *t*-test). (B–D) Effect on endogenous HP1β or ASH1L amount at the ectopic integration site of alphoid^tetO^ by tethering of ASH1L or HP1s. (B) Schematic of the assay. (C) Representative images of assembly on alphoid DNA. Yellow square areas indicate the integration sites that are shown magnified as inset images. (D) Percentage of cells exhibiting immunodetection of endogenous HP1β or ASH1L assembly on ectopic alphoid^tetO^ DNA integration sites at 1 d after transfection of tetR–EYFP–ASH1L^1901–2964^ or tetR– EYFP–HP1β expression plasmids. More than 100 cells were counted for each assay. Results are mean±s.e.m. (*n*=3 experiments). (E) Influence of ASH1L or HP1β tethering on the chromosome (HAC) stability. HACs were visualized as tetR–EYFP fluorescence spots. Initially, the tetR–EYFP fusion-expressing alphoid^tetO^ HAC-bearing cells were cultured in the presence of doxycycline (without tethering condition). The tetR–EYFP-fused protein was then tethered to HAC by washing doxycycline out. A time course experiment after doxycycline washout (6 h to 1 week) was performed. HACs were counted as the number of tetR–EYFP spots. Gray, orange and blue bars indicate the percentage of cells containing ≥2, 1 or 0 HACs, respectively. More than 50 cells with detectable EYFP signal were analyzed for each assay. Results are mean±s.e.m. (*n*=3 experiments). Scale bars: 5 µm.

First, we confirmed the effect of ASH1L tethering on H3 modification state at the ectopic alphoid DNA integration site using ChIP analysis. Tethering of tetR–EYFP–ASH1L resulted in increased H3K36me2/3 on the alphoid^tetO^ integration site, whereas levels of H3K9me3 and H3K27me2 decreased (Fig. 5A). Thus, in response to ASH1L tethering, H3K36me2/3 was inversely correlated with H3K9me3 or H3K27me2. HP1 binds to H3K9me3 (Nakayama et al., 2001; Bannister et al., 2001; Lachner et al., 2001).

Next, we determined the effect of tethering tetR–EYFP–HP1β or tetR–EYFP–ASH1L to the ectopic alphoid^tetO^ array on the localization of endogenous ASH1L or HP1β. Tethering tetR–EYFP–HP1β reduced the binding of endogenous ASH1L, and vice versa (endogenous ASH1L or HP1β were reduced by 80%) (Fig. 5B–D). It thus appears that ASH1L and HP1β assembly might be mutually exclusive on alphoid DNA.

We then examined effects of tethering ASH1L or HP1β on centromere function. We measured the mitotic stability of the alphoid^tetO^ HAC tethered with tetR–EYFP–ASH1L or tetR–EYFP–HP1β by counting EYFP HAC signals per nucleus (Fig. 5E). After tethering the control tetR–EYFP construct for 1 week, less than 8% of nuclei lacked a HAC signal (HAC=0) and over 83% of nuclei maintained one copy of the HAC per nucleus (HAC=1). In contrast, upon tetR–EYFP–HP1β tethering 28.8% of nuclei lost the HAC signal. Similarly, 47.1% of nuclei lost the HAC signal after tetR–EYFP–ASH1L tethering. It therefore appears that unbalanced assembly of ASH1L or HP1β on the HAC impairs centromere function.

### Overexpression of heterochromatin factors in ASH1L-depleted cells reduces chromosomal stability

**Fig. 6.**
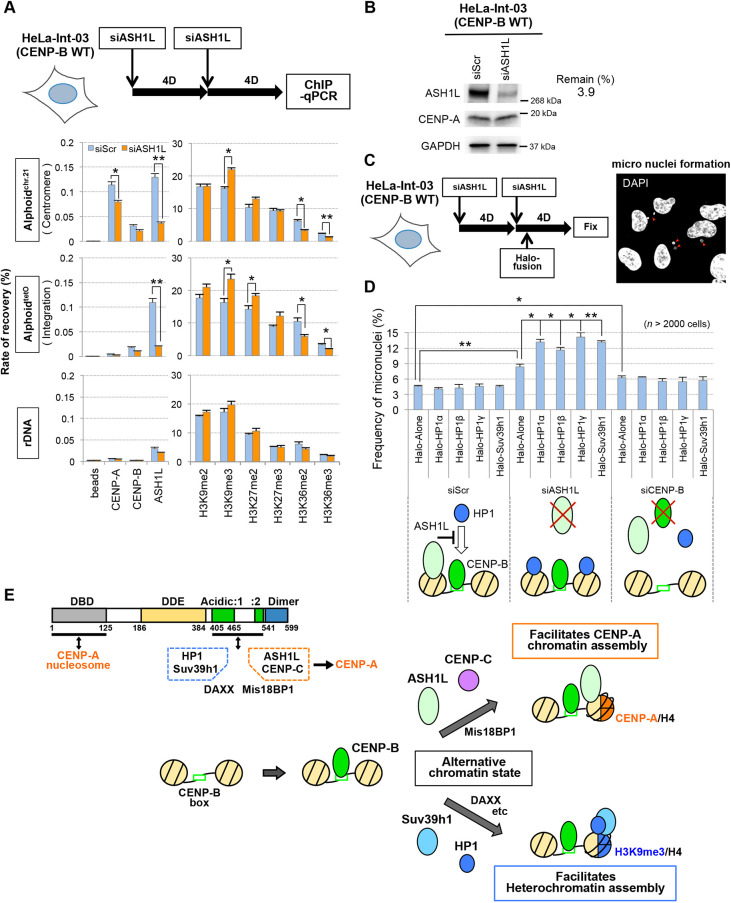
**Evaluation of ASH1L function at the centromere.** (A) ChIP assay of ASH1L-depleted cells. ASH1L siRNA or control scramble siRNA was transfected twice (8 and 4 d before cell harvest) into HeLa-Int-03 cells. Results are mean±s.e.m. (*n*=3 experiments). *P<0.05, **P<0.01 (two-tailed *t*-test). (B) Evaluation by immunoblotting of ASH1L depletion in the cells used in A. The ASH1L-knockdown level shown on the right was calculated from a dilution series of whole cell extract (Fig. S5A). Total protein amounts were adjusted using the GAPDH expression level. (C,D) Perturbation of the balance between ASH1L and HP1s increases the frequency of micronuclei formation. HeLa-Int-03 cells were transfected with siRNA (scramble, ASH1L or CENP-B) and plasmids expressing the indicated Halo-tagged proteins. The plasmid transfection was performed 5 d after the first siRNA transfection. Cells were stained with DAPI and scored for micronuclei formation. Red arrowheads in the representative image indicate micronuclei. More than total 2000 cells were analyzed for each assay. Results are mean±s.e.m. (*n*=3 experiments). *P<0.05, **P<0.01 (two-tailed *t*-test). Lower scheme represents the action of different proteins on centromeric alphoid DNA in this experiment. (E) A model for the effect of CENP-B. Top left: relationship between CENP-B domains, assembling proteins and *de novo* CENP-A assembly. Bottom right: changes in chromatin state on alphoid DNA facilitated by CENP-B binding. CENP-B stabilizes kinetochore function by interactions with CENP-A nucleosomes via the DBD and with CENP-C via the acidic domain. Additionally, Mis18BP1 assembly on alphoid DNA is facilitated by the CENP-B acidic domain. Mis18BP1 interacts with CENP-C. CENP-B may promote competent chromatin for CENP-A assembly via both the DBD and acidic domain. ASH1L assembly at the centromere is promoted by the CENP-B acidic domain. Similarly, Suv39h1 and HP1 assembly is facilitated by the CENP-B acidic domain. ASH1L has activity that facilitates CENP-A assembly. Conversely, Suv39h1 and HP1 suppress CENP-A assembly.

We investigated whether ASH1L depletion affects the chromatin status of alphoid DNA in HeLa cells using ChIP analysis. ASH1L siRNA treatment reduced total cellular ASH1L levels to 3.9% (Fig. 6B). ASH1L enrichment on the chromosome 21 centromeric alphoid DNA (alphoid^chr.21^) and the ectopic alphoid^tetO^ array were both significantly decreased by ASH1L depletion (∼69% and 79%, respectively) (Fig. 6A). We also quantified H3 modifications, and detected a significant decrease in H3K36me2/3 levels and increased H3K9me3 and H3K27me2 levels following ASH1L depletion. Thus, the ASH1L depletion results showed the opposite effects to the effects of ASH1L tethering shown in Fig. 5A, and the effects were consistent. Interestingly, CENP-A levels on the centromeric alphoid^chr.21^ significantly decreased by 27% in ASH1L-depleted HeLa cells.

Next, we examined the effects of perturbing the balance of ASH1L and HP1s on natural chromosomes using siRNA depletion of ASH1L followed by overexpression of heterochromatin proteins. We then scored for the presence of micronuclei and lagging chromosomes (Fig. 6C,D). Five days after transfection with ASH1L siRNA, cells were transfected with a plasmid expressing Halo-tagged heterochromatin proteins HP1α, HP1β, HP1γ or Suv39h1. In cells treated with control scrambled siRNA (siScr), the frequency of micronuclei and lagging chromosomes was not significantly increased by overexpression of the heterochromatin proteins (control Halo-tag alone, 4.6%; Halo–HP1α, 4.1%; Halo–HP1β, 4.2%; Halo–HP1γ, 4.6%; and Halo–Suv39h1, 4.5%). In contrast, after ASH1L depletion, the frequency of micronuclei was increased roughly twofold upon expression of the control Halo-tag alone (8.4%). Overexpression of Halo-tagged heterochromatin proteins further increased the micronucleus frequency (Halo–HP1α, 13.2%; Halo–HP1β, 11.6%; Halo–HP1γ, 14.1%; and Halo–Suv39h1, 13.2%). In CENP-B-depleted cells, the micronucleation frequency was slightly but significantly increased even by the control Halo-tag alone (6.3%), but not further increased by overexpressing Halo-tagged heterochromatin proteins. This can be explained by the lack of recruitment of heterochromatin proteins by CENP-B, which consequently prevents heterochromatin from being excessively assembled into centromeres.

Taken together, these results suggest that ASH1L may act on CENP-B chromatin to suppress the excessive assembly of HP1 and heterochromatin factors. Conversely, HP1 acts on CENP-B chromatin to suppress excessive assembly of ASH1L (Fig. 5A–D). It thus appears that CENP-B and these recruiting factors create a ‘bidirectional’ alternative epigenetic chromatin balance that promotes accurate chromosome segregation (Fig. 6E).

### CENP-B affects the methylation-level of histone H3 on alphoid DNA in HT1080 cells

In this study, HeLa cells were used to demonstrate the balance of CENP-B-dependent ASH1L and HP1β assembly on alphoid DNA. However, most previous studies, including ours, have used human fibrosarcoma cells (HT1080) to demonstrate *de novo* HAC formation and *de novo* CENP-A assembly with the introduced alphoid DNA. Therefore, we confirmed the CENP-B-dependent histone modification balance using alphoid DNA introduced into HT1080 cells. HT1080 cells exhibit much less heterochromatin assembly activity on alphoid DNA than HeLa cells (Ohzeki et al., 2012).

**Fig. 7.**
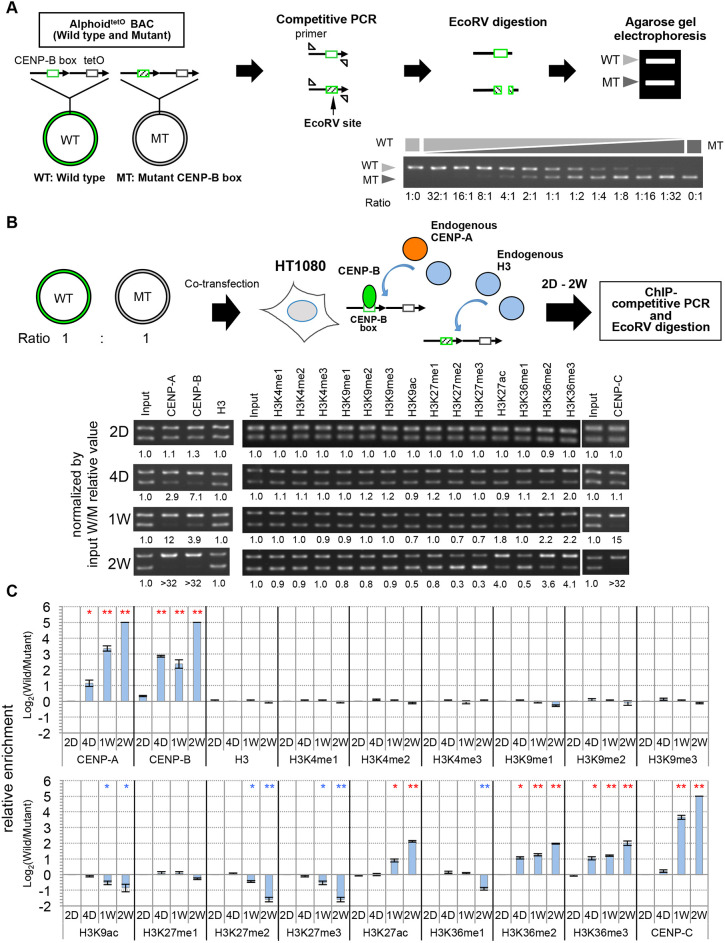
**Detection of CENP-B-dependent histone modification on introduced alphoid DNA.** Exploration of CENP-B-induced histone H3 modification on introduced alphoid^tetO^ DNA using ChIP and competitive PCR. (A) Example of competitive PCR control assay. The wild-type and mutant CENP-B box alphoid^tetO^ BAC DNAs were mixed at the indicated ratios and amplified by PCR with the same primer set (for alphoid^tetO^ DNA-specific amplification). The PCR products were amplified keeping the initial ratio, then digested with EcoRV and analyzed by agarose gel electrophoresis. The light and dark gray arrowheads indicate the PCR fragment from the wild type CENP-B box alphoid^tetO^ DNA and the mutant CENP-B box alphoid^tetO^ DNA, respectively. (B) Upper panel: schematic of the ChIP and competitive PCR. HT1080 was co-transfected with both wild-type and mutant CENP-B box alphoid^tetO^ BAC DNA. Lower panel: CENP-B-dependent *de novo* CENP-A assembly. After 2 d (2D), 4 d (4D), 1 week (1W), and 2 weeks (2W) of transfection, cells were analyzed with antibodies against CENP-A, CENP-B, CENP-C, histone H3 (H3) and the indicated H3 modifications. The relative enrichment of the wild-type CENP-B box alphoid^tetO^ DNA versus the mutant CENP-B box alphoid^tetO^ DNA is shown below the gel images. (C) The relative enrichment of recovered alphoid^tetO^ DNAs (wild-type or mutant CENP-B box) are displayed as log_2_ enrichment. Results are mean±s.e.m. (*n*=3 experiments). *P<0.05, **P<0.01 (two-tailed *t*-test). Red and blue asterisks indicate significant increased or decreased WT:MT ratio, respectively.

A preliminary experiment showed that robust CENP-A and CENP-B assembly activities with introduced wild-type CENP-B box alphoid^tetO^ DNA (which can bind to CENP-B) were detected at 2 weeks after transfection using ChIP-qPCR in HT1080 cells (Fig. S4A). Conversely, mutant CENP-B box alphoid^tetO^ DNA (which does not bind to CENP-B) did not show either CENP-A or CENP-B assembly. We therefore analyzed the H3 modification status in parallel with CENP-A chromatin formation on transfected alphoid DNA bearing either wild-type or mutant CENP-B boxes using a sensitive ChIP–competitive PCR analysis (Fig. 7B,C) at early time points after co-transfection (Ohzeki et al., 2002; Okada et al., 2007). The competitive PCR can accurately compare the abundance ratio between wild-type and mutant CENP-B box alphoid DNA (Fig. 7A).

Relative enrichment of CENP-A was detected on the wild-type CENP-B box alphoid^tetO^ DNA 4 days after transfection (Fig. 7B,C). CENP-C assembly was also detected on the wild-type CENP-B boxes with slower kinetics at 1 week after transfection. Analysis in parallel also detected a comparatively early relative enrichment for H3K36me2 and H3K36me3 on the wild-type CENP-B boxes, within 4 days after transfection. A similar, albeit delayed, enrichment for H3K27ac was also detected at 1–2 weeks after transfection. Relative enrichments of H3K27me2 and me3, which have been reported to be mutually exclusive with H3K36 methylation (Yuan et al., 2011), were detected on the mutant CENP-B box alphoid^tetO^ DNA 1–2 weeks after transfection (Fig. 7B,C). These results suggest that CENP-B-dependent methylation status changes occur earlier for H3K36me2/3 than for H3K27me2/3. In addition, these changes may include demethylation as well as methylation, because several demethylases were also identified in Fig. 1. These results can be explained if assembly of ASH1L or similar factors on alphoid DNA requires the presence of CENP-B. Indeed, we confirmed ASH1L localization at the centromere in HT1080 cells (Fig. S4B), and dominant assembly of ASH1L on transfected alphoid DNAs with CENP-B boxes continued for at least 2–4 weeks (Fig. S4C,D).

### The CENP-B acidic domain and DNA-binding domain both assemble CENP-A *de novo* on exogenous alphoid DNA in HT1080

Fig. 3 shows that the CENP-B acidic domain recruits various factors, including ASH1L. Thus, we decided to investigate the involvement of the acidic domain on *de novo* CENP-A assembly. To analyze the mechanisms by which CENP-B promotes *de novo* centromere formation, we attempted to identify CENP-B domains involved in CENP-A chromatin formation on transfected alphoid DNA. However, this was complicated because the CENP-B N-terminal DNA-binding domain (DBD) is both essential for CENP-B box binding and also interacts with CENP-A nucleosomes to induce *de novo* CENP-A assembly (Okada et al., 2007; Fujita et al., 2015).

**Fig. 8.**
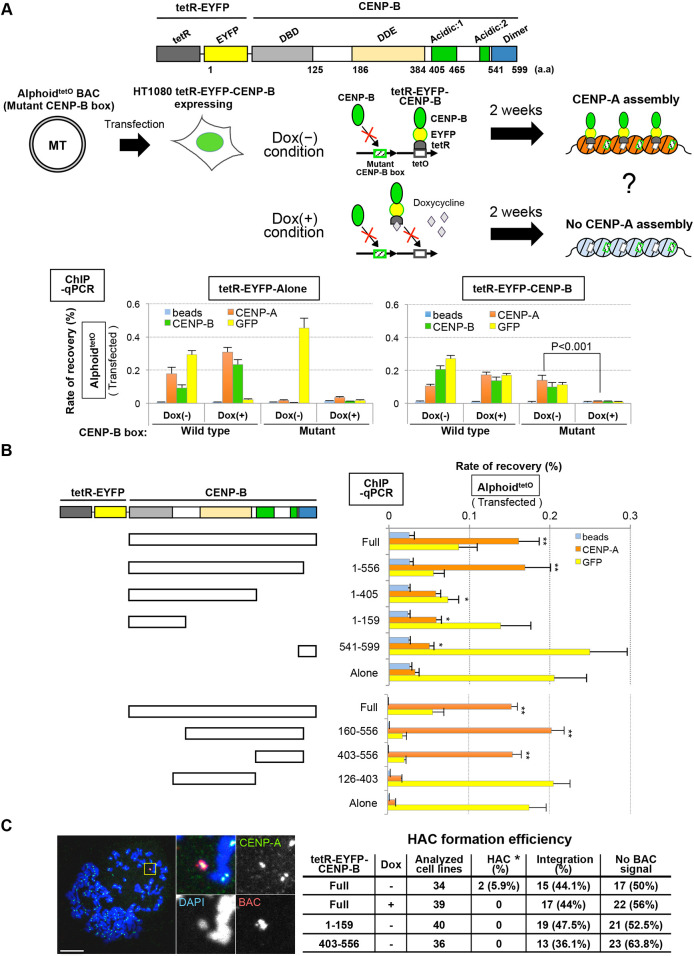
**Identification of a *de novo* CENP-A assembly domain on CENP-B.** (A) Functional verification of tetR–EYFP–CENP-B fusions. Top: schematic of tetR–EYFP–CENP-B construction. Middle: schematic of the experimental design and expected effect of tetR–EYFP–CENP-B. Bottom: effect on CENP-A assembly by tethering of tetR–EYFP–CENP-B or tetR–EYFP alone (negative control). The cells were cultured in the presence (+) or absence (−) of doxycycline (Dox) during 2 weeks (2W) post-transfection. Enrichment of tetR-fusion proteins, CENP-A and CENP-B on transfected alphoid^tetO^ DNAs was detected by ChIP-qPCR analysis using the indicated antibodies: blue, without antibody for negative control; orange, anti-CENP-A; green, anti-CENP-B; yellow, anti-GFP antibody for EYFP. Results are mean±s.e.m. (*n*=3 experiments). *P*-values are indicated in the figure (two-tailed *t*-test). (B) Identification of CENP-B domains enhancing *de novo* CENP-A assembly. Left: schematic of the tetR-fusion CENP-B domain series. Right: the CENP-A assembly activity of each CENP-B domain was detected using ChIP-qPCR in the absence of Dox. Results are mean±s.e.m. (*n*=3 experiments). *P<0.05; **P<0.005 (two-tailed *t*-test). (C) HAC formation by tethering of CENP-B domain series fusions to mutant CENP-B box alphoid^tetO^ BAC DNA in an HT1080 cell line expressing tetR–EYFP–CENP-B. Left: mitotic chromosomes from one of the HAC cell lines obtained (HT1080 N21-1, see below) were stained with DAPI (blue), anti-CENP-A antibody (green), and BAC FISH probe (red). Scale bar: 10 µm. Right: table showing HAC formation efficiency. Naked mutant CENP-B box alphoid^tetO^ BAC DNAs were transfected into HT1080 cell lines stably expressing tetR–EYFP fusion proteins indicated in the table (see Materials and Methods) in the presence or absence of doxycycline. The ‘analyzed cell lines’ column shows the number of colonies of cells isolated under selection using G418. The fate of the introduced BAC DNA was analyzed by FISH using a BAC probe. BAC DNA was detected either as an entity independent from the host chromosome (HAC) or as part of the host chromosome (integration). Two HAC cell lines were obtained. In these two cell lines, HAC signals were detected as a single HAC per cell in 92.3% and 90% of cells (*n*>20 cells). No integration signal was observed on the host chromosomes in these two HAC cell lines.

To bypass the requirement for CENP-B DBD–CENP-B box interaction, we tethered tetR-fused CENP-B to synthetic alphoid DNA containing a tetO sequence (alphoid^tetO^ DNA). Bacterial artificial chromosomes (BACs) containing alphoid^tetO^ DNA arrays carrying the wild-type or mutant CENP-B boxes were introduced into HT1080 cells stably expressing tetR–EYFP (control) or tetR–EYFP–CENP-B in the presence or absence of doxycycline (Dox+, no tethering; Dox−, tethering) (Fig. 8A). CENP-A assembly was detected on wild-type CENP-B box alphoid^tetO^ DNA in cell lines expressing either tetR–EYFP or tetR–EYFP–CENP-B in the presence or absence of doxycycline. This assembly reflects interactions between the endogenous CENP-B DBD and CENP-B box DNA.

In contrast, mutant CENP-B box alphoid^tetO^ DNA failed to assemble CENP-A in cells expressing either tetR–EYFP or tetR–EYFP–CENP-B in the presence of doxycycline. However, when doxycycline was absent (allowing tetR binding to the tetO sequences), CENP-B and CENP-A assembly were readily detected on the alphoid^tetO^ DNA (Fig. 8A). Thus, CENP-B tethering to the alphoid^tetO^ DNA via the tetR–tetO interaction can bypass the requirement for CENP-B boxes in *de novo* CENP-A assembly.

Next, we used the tetR tethering assay to assess CENP-A assembly activity promoted by various CENP-B sub-domains. Mutant CENP-B box alphoid^tetO^ DNAs were transfected into cell lines stably expressing tetR–EYFP fused to a series of CENP-B deletions. Significant CENP-A assembly was detected with fusions carrying the N-terminal DBD and also fusions carrying the acidic domains (amino acids 403–556) (Fig. 8B). CENP-A assembly activity was low following tethering of the CENP-B dimer domain, but significantly above background. This is likely explained by interactions with endogenous full-length CENP-B (Fig. 3B) (Kitagawa et al., 1995). Thus, both the DBD and acidic domain of CENP-B can promote *de novo* CENP-A assembly on transfected alphoid DNA.

A HAC formation assay (Ikeno et al., 1998; Ohzeki et al., 2002) confirmed that CENP-A assembly via CENP-B tethering can induce functional centromere formation on exogenous mutant CENP-B box alphoid^tetO^ DNA. When transfections were done in the absence of doxycycline, HACs were formed in two of 34 transformed HT1080 cell lines expressing tetR–EYFP–CENP-B^Full^ (5.9%). In contrast, in the presence of doxycycline, no HAC was detected in 39 cell lines (Fig. 8C). No HACs were detected in HT1080 cell lines expressing either tetR–EYFP–CENP-B^DBD^ or tetR–EYFP–CENP-B^acidic domain^. Thus, tetR-fused, full-length CENP-B tethered to mutant CENP-B box alphoid DNA via tetO retains the ability to assemble *de novo* CENP-A competent for functional centromere formation, but either the N-terminal DBD or the acidic domain alone does this with reduced effectiveness. Further analysis is needed to determine whether either domain alone has the ability to assemble functional centromeres at low efficiency.

Furthermore, to investigate whether ASH1L is involved in the *de novo* CENP-A assembly pathway promoted by the CENP-B acidic domain in HT1080 cells (Fig. 8B), we depleted ASH1L and used ChIP-qPCR to quantify CENP-A assembly following tethering of tetR–EYFP–CENP-B^403–556^ to alphoid^tetO^ DNA with mutant CENP-B boxes. Levels of *de novo* CENP-A assembly and H3K36 methylation on transfected mutant CENP-B box alphoid^tetO^ DNA were significantly decreased in the ASH1L-depleted cells (Fig. S5B). Since anti-CENP-B antibody showed no enrichment of mutant CENP-B box alphoid^tetO^ DNA, it is obvious that endogenous CENP-B is not involved in this CENP-A assembly reaction. Importantly, no significant change was detected in total cellular CENP-A levels following ASH1L depletion to 6.3% of control levels (Fig. S5A). These results suggest that ASH1L is critical also for *de novo* CENP-A assembly via the CENP-B acidic domain in HT1080.

## DISCUSSION

CENP-B is a remarkable protein that can promote ‘open’ or ‘closed’ chromatin states on alphoid DNA, with profound consequences for centromere activity and kinetochore assembly. Here, we have shown that two independent CENP-B domains can stimulate *de novo* CENP-A assembly on introduced alphoid DNA. In addition to the N-terminal DNA-binding domain, which can bind to CENP-A nucleosomes (Fujita et al., 2015; Fachinetti et al., 2015), the CENP-B acidic domain (amino acids 403–556; Earnshaw, 1987b) facilitated assembly of the H3K36 methyltransferase ASH1L, thereby inducing an open chromatin state competent for CENP-A assembly or exchange. Thus, the CENP-A assembly mechanism involving the acidic domain is quite different from that involving the DBD. Importantly, full-length CENP-B containing both domains could efficiently induce functional centromere and HAC formation on transfected alphoid DNA.

### Functionally antagonistic histone modifications induced by CENP-B binding

We previously reported that CENP-B facilitates H3K9 methylation at an ectopic alphoid DNA integration site on chromosome arms in MEF cell lines (Okada et al., 2007). Here, we have confirmed that CENP-B binding promotes Suv39h1 and HP1 assembly and H3K9 methylation on alphoid DNA at both ectopic integration sites and endogenous centromeres in human cell lines. Importantly, CENP-B-induced H3K9 methylation only occurs subsequent to CENP-A and CENP-C assembly on exogenous alphoid DNA (Ohzeki et al., 2012). Remarkably, H3K36 methylation, a mark associated with active transcription, was also found on alphoid DNA arrays and occurred with similar timing to CENP-A and CENP-B assembly. Our results demonstrate that ASH1L and/or HP1 recruited by the CENP-B acidic domain assemble mutually exclusively and dynamically on alphoid DNA to ‘bidirectionally’ generate alternative epigenetic chromatin, whose balance is important to maintain centromere function (Figs 4-6).

CENP-B binding to the CENP-B box is inhibited by DNA methylation, which is typically associated with transcriptionally inactive chromatin (Tanaka et al., 2005; Okada et al., 2007). Centromeric chromatin states might be dynamic and/or exchangeable with each other during cell cycle progression, differentiation or senescence. Suv39h1 expression levels and H3K9me3 levels on chromosomes in HeLa cells are much higher than those in HT1080 cells (Ohzeki et al., 2012). Interestingly, levels of CENP-B at centromeres also differs between cell types and during senescence. Moreover, the balance of heterochromatin formation at centromeres, as revealed by HP1 and CENP-A accumulation, can change as human embryonic fibroblast cells undergo senescence (Maehara et al., 2010).

Antagonistic ‘open’ and ‘closed’ histone modifications could be distributed on different sub-regions of the long alphoid DNA repeats. Indeed, alphoid DNA sub-regions forming centromere chromatin can vary on the same chromosome (as observed for human chromosome 17; Maloney et al., 2012). Mapping the chromatin state of sub-regions of the alphoid DNA is challenging due to the highly repetitive nature of the DNA. For better understanding of the correlation between the centromeric chromatin states and kinetochore assembly, it is important to clarify the mechanisms by which CENP-B binding to specific repetitive DNA structures can produce such radically different functional chromatin states.

### The CENP-B acidic domain creates a bidirectional chromatin ‘switch’

Our screening identified a number of factors recruited to chromatin by the CENP-B acidic domain (CENP-A was unusual, in that it was also recruited by the DBD). How do acidic domains recruit such various factors to chromatin? We previously suggested that acidic regions might unfold chromatin to allow access of other proteins, interact with histones to recruit other associated factors to chromatin or directly interact with other chromatin proteins (Earnshaw, 1987b). Indeed, many histone chaperones have acidic domains, and subsequent studies have supported the proposal that interaction with basic histone residues is important to promote proper association of histones and DNA (Tyler, 2002). The histone chaperone FACT affects nucleosome structure by interaction between its acidic domain and histones (Tsunaka et al., 2016). The CENP-B acidic domain promotes H3.3 assembly on alphoid DNA (Shono et al., 2015; Morozov et al., 2017; and data not shown), suggesting that it produces a chromatin state competent for histone replacement. This is consistent with older studies in which polyglutamic acid was able to promote nucleosome assembly from isolated histones *in vitro* (Stein et al., 1979). It is possible that, in addition to modifying chromatin structure, targeting of various chromatin-related factors might also involve direct interactions with the acidic domain. In future studies, it will be important to map interactions between the CENP-B acidic domain and client proteins by structural analysis.

Chromatin modifications induced by the CENP-B acidic domain promote structural changes that effectively produce a ‘bidirectional’ state of alphoid DNA chromatin. On the one hand, CENP-B can facilitate CENP-A assembly by recruiting ASH1L and other centromere-related proteins or by replacing histones – for example, with H3.3. H3.3 has been suggested to be an important placeholder for CENP-A replenishment, but is also important for maintenance of heterochromatin (Dunleavy et al., 2011; Shono et al., 2015; Múller and Almouzni, 2017). On the other hand, CENP-B facilitates heterochromatinization in non-centromeric regions (especially at the ectopic alphoid DNA integration site or pericentromere, that is, in the absence of the epigenetic CENP-A mark) by recruiting Suv39h1 and HP1. These results suggest that CENP-B can switch its activity according to the presence or absence of the functional centromere, possibly because the CENP-B DBD interacts with CENP-A nucleosomes.

H3.3 localization is particularly prominent on transcriptionally active chromatin (Mito et al., 2005). Centromeric transcription occurs at low levels, even during mitosis (Chan et al., 2012; Molina et al., 2016). This centromeric transcription is important for centromere function and assembly (Wong et al., 2007; Quénet and Dalal, 2014; Rošić et al., 2014; Chen et al., 2015; Molina et al., 2016; McNulty et al., 2017; Bobkov et al., 2018), and excess methylation of H3K27 inhibits both transcription and CENP-A assembly (Martins et al., 2016). It is possible that this transcription determines the direction of the CENP-B chromatin ‘switch’.

H3.3 recruitment and H3K36 methylation downstream of ASH1L recruitment by the CENP-B acidic domain might promote transcriptional activity and subsequent CENP-A assembly. Alternatively, because H3K36me2/3 typically arises downstream of transcription, centromeric transcription promoted by another mechanism might promote interactions between CENP-B and its associated factors to create the open state of centromere chromatin that is required for kinetochore assembly and stability. It is possible that as-yet-undiscovered functional interactions between CENP-B and the RNA polymerase II machinery might be at the heart of the bidirectional ability of CENP-B to promote heterochromatic or transcriptionally active chromatin states.

We conclude that CENP-B regulates CENP-A chromatin not only by interacting with CENP-A and CENP-C, but also by recruiting histone modifiers. Future studies will explore how histone modifications associated with contrasting chromatin states interact with each other on alphoid DNA sub-regions and how this bidirectional chromatin ‘switch’ is controlled by CENP-B and other centromere-specific factors.

## MATERIALS AND METHODS

### Cell lines

Human cultured cells, HT1080, HeLa, HeLa-HAC-2-4 (containing the alphoid^tetO^ HAC) and HeLa-Int-03 (containing the ectopic alphoid^tetO^ DNA integration site) were described previously (Shono et al., 2015; Ohzeki et al., 2012, 2016). HT1080 stably expressing tetR–EYFP or tetR–EYFP–CENP-B fusions [full length (N21), 1–556, 1–405, 1–159, 160–556, 403–556, 126–403, or 541–599], and HeLa stably expressing tetR–EYFP or tetR–EYFP–CENP-B fusions (full length, 1–159, 126–403, 403–556, 541–599) cell lines were established using the Jump-in integration system (Life Technologies) with pJETY3 and pJTI PhiC31 plasmids (expression for tetR–EYFP-fused gene and Phi-C31 integrase, respectively). The HeLa-Int-03-CENP-B knockout cell line was established using CRISPR/Cas9. For the knockout of CENP-B genes, we constructed pJ4IB-Cas9 and pU6CR-CENP-B [for expression of Cas9 protein and guideRNA against the CENP-B gene (5′-GAAGAACAAGCGCGCCATCC-3′)] vectors, and these vectors were co-transfected into HeLa-Int-03. In isolated single clones, CENP-B gene deletions at all the alleles were confirmed by sequencing of genomic PCR products using a MiSeq sequencer (Illumina).

### Cell culture and transfection

HT1080, HeLa and their derivative cells were cultured in Dulbecco's Modified Eagle's medium (Wako; 043-30085), supplemented with 10% tet-approved FBS (Clontech; 631106), at 37°C in a 5% CO_2_ atmosphere. To dissociate tetR–EYFP-fused protein from the tetO sites, doxycycline was added to the medium at a concentration of 1 ng/µl. For transfection of plasmids or siRNA, FuGENE HD (Promega; E2312) or Lipofectamine2000 (ThermoFisher; 11668027) was used. siRNA oligos (Silencer^®^ select siRNAs) for CENP-B (s2909), ASH1L (s31702) and negative control (4390844) were obtained from ThermoFisher.

### Cell staining and Halo-fused protein labeling

Cells were fixed with 80% acetone at −20°C for 10 min or 2.5% formaldehyde (Wako; 063-04815) at room temperature for 10 min. Formaldehyde-fixed cells were permeabilized with 0.5% Triton X-100 in PBS. Fixed cells were blocked in 2% BSA in PBS for 30 min. Cells were incubated at 37°C for 1 h with each of the primary and secondary antibodies. For chromosome spreads, HT1080 tetR–EYFP–CENP-B N21 cell line (Fig. 8C) was treated with 350 nM TN-16 (Wako) (Kitagawa et al., 1995) for 3 h in the culture medium. Mitotic cells were harvested, incubated in 0.075 M KCl for 10 min on ice, and then spread on a cover-glass using a Cytospin3 centrifuge (Shandon). The subsequent immunostaining and fluorescence *in situ* hybridization (FISH) were performed to confirm HAC formation according to a previously reported method (Ikeno et al., 1998; Ohzeki et al., 2002). For Halo-tag labeling, cells were cultured in the presence of 10 nM Halo-TMR-Ligand (Promega; G8251) or 10 nM Halo-Biotin-Ligand (Promega; G8281).

### Immunoblotting

Immunoblotting was performed as previously described (Shono et al., 2015) with slight modifications. To separate and detect ASH1L protein, whole cell extracts were electrophoresed using a Multi Gel II mini 5 system (CosmoBio; 443138). HiMark™ Pre-Stained protein standard (ThermoFisher; LC5699) was used as molecular weight marker.

### Microscopy and quantification of images

For quantification analysis of CENP-A (Fig. 2), *z*-stack images were acquired with a spacing of 0.22 µm to cover an entire nuclear signal on an Axio Observer.Z1 microscope (Zeiss) equipped with a CSU-X1 confocal scanner unit (Yokogawa), iXon3 DU897E-CS0 camera (Andor) and Plan-Apochromat 100×/1.46 oil lens (Zeiss) using Andor iQ2 software (Andor). Quantitative analysis of acquired images was performed as previously described (Shono et al., 2015). Other cell images were acquired on an Axio Observer.Z1 (Zeiss) equipped with a LSM700 scanning module and an Objective Plan-Apochromat 63×/1.46 oil lens (Zeiss) using ZEN 2009 software (Zeiss). For quantification analysis in Fig. 4, *z*-stack images were acquired with a spacing of 0.36 µm, and with total depth of 5.4 µm. Signal intensity was acquired for each pixel in a 22×22 pixel region around the spot from a *z*-stack maximum intensity projection cell image using Fiji software (Schindelin et al., 2012). The pixel intensities located in a symmetric position with respect to the *x*-axis and *y*-axis were summed up. The integrated pixel intensities at the same distance from the spot were averaged.

### ChIP assay and qPCR or competitive PCR

The ChIP assay was performed as previously described (Fujita et al., 2015) with slight modifications. The cells were trypsinized, harvested in a centrifuge tube, washed once with PBS, and fixed in 1.0% formaldehyde (Sigma; F8775) at 25°C for 15 min. After fixation, the cells were suspended in sonication buffer, containing 20 mM Tris-HCl (pH 8.0), 0.5 mM EDTA, 0.5× cOmplete™ Protease Inhibitor Cocktail (PIC) (from a 50× stock solution made of one tablet dissolved in 5 ml PBS; Sigma-Aldrich; 5056489001), 1 mM DTT, 40 µM MG132 and 0.02% SDS and then sonicated with a Bioruptor (Cosmo Bio; UCD-300) to generate an average DNA size of 0.5–1 kb at power level H for 30–40 min with 30 s ON/OFF intervals. After the sonication, the soluble chromatin (as input) was diluted with an equivalent volume of IP buffer containing 20 mM Tris-HCl (pH 8.0), 300 mM NaCl, 0.5 mM EDTA, 0.5× PIC, 40 µM MG132, 1 mM DTT, 0.05% SDS, 1% Triton X-100, and 5% glycerol, then immunoprecipitated using antibody with Dynabeads–Protein G (Life Technologies; 10003D) or Protein G Sepharose 4 Fast Flow (GE Healthcare; 17061801). The immunoprecipitated fraction was washed three times with IP buffer, and incubated to denature DNA/Protein fixation as follows; with RNaseA (ThermoFisher; EN0531) at 37°C for 1 h, with Proteinase K (Sigma-Aldrich; 3115836001) at 55°C for 2 h, then with additional incubation at 65°C for 12 h. The ChIP DNA was purified using a MinElute PCR purification kit (Qiagen). The DNA was quantified by real-time PCR using SYBR Premix EX taqII (Takara Bio; RR820S) and the following primer sets: tetOF and tetOR for alphoid^tetO^ DNA (Fujita et al., 2015); 21-I alphoid a (21alpF and 21alpR) for the 11mer of chromosome 21 alphoid DNA (Ohzeki et al., 2012); and 5SrDNA-F1 and 5SrDNA-R1 for ribosomal DNA (Nakano et al., 2003). In competitive PCR assays, amplified DNAs were digested with EcoRV and then visualized by electrophoresis on 2.5% agarose gel (Okada et al., 2007).

### Antibodies

For ChIP assays and immunostaining, we used anti-CENP-A (1 μg for ChIP, 1:1000 dilution for immunostaining; monoclonal antibody A1; Ohzeki et al., 2002), anti-CENP-B (1 μg for ChIP, 1:1000 dilution for immunostaining; monoclonal antibody 5E6C1, which recognizes the CENP-B C-terminal dimer domain; Ohzeki et al., 2002), anti-CENP-C (3 μl for ChIP; MBL; DP030), anti-ASH1L (3 μl for ChIP, 1:3000 dilution for immunostaining; Bethyl; A301-749A), anti-HP1β (Cell Signaling, 8676S for ChIP; 2μl; Abcam, ab10811 for immunostaining; 1:100 dilution), anti-H3 (1 μg for ChIP; MABI; 0301), anti-H3K4me1 (1 μg for ChIP; MABI; 0302), anti-H3K4me2 (1 μg for ChIP; MABI; 0303), anti-H3K4me3 (1 μg for ChIP; MABI; 0304), anti-H3K9me1 (1 μg for ChIP; MABI; 0306), anti-H3K9me2 (1 μg for ChIP; MABI; 0307), anti-H3K9me3 (1 μg for ChIP; MABI; 0308), anti-H3K9ac (1 μg for ChIP; MABI; 0305), anti-H3K27me1 (1 μg for ChIP; MABI; 0321), anti-H3K27me2 (1 μg for ChIP; MABI; 0324), anti-H3K27me3 (1 μg for ChIP; MABI; 0323), anti-H3K27ac (1 μg for ChIP; MABI; 0309), anti-H3K36me1 (1 μg for ChIP; MABI; 0331), anti-H3K36me2 (1 μg for ChIP; MABI; 0332) and anti-H3K36me3 (1 μg for ChIP; MABI; 0333) antibody.

## Supplementary Material

Supplementary information

Reviewer comments
