# Supplementary material for: CENP-B creates alternative epigenetic chromatin states permissive for CENP-A or heterochromatin assembly
Source: J Cell Sci. 2020 Aug 11;133(15):jcs243303. doi: 10.1242/jcs.243303 (PMC7438015; doi:10.1242/jcs.243303)
Supplement: Supplementary information [file joces-133-243303-s1.pdf]

Figure S1

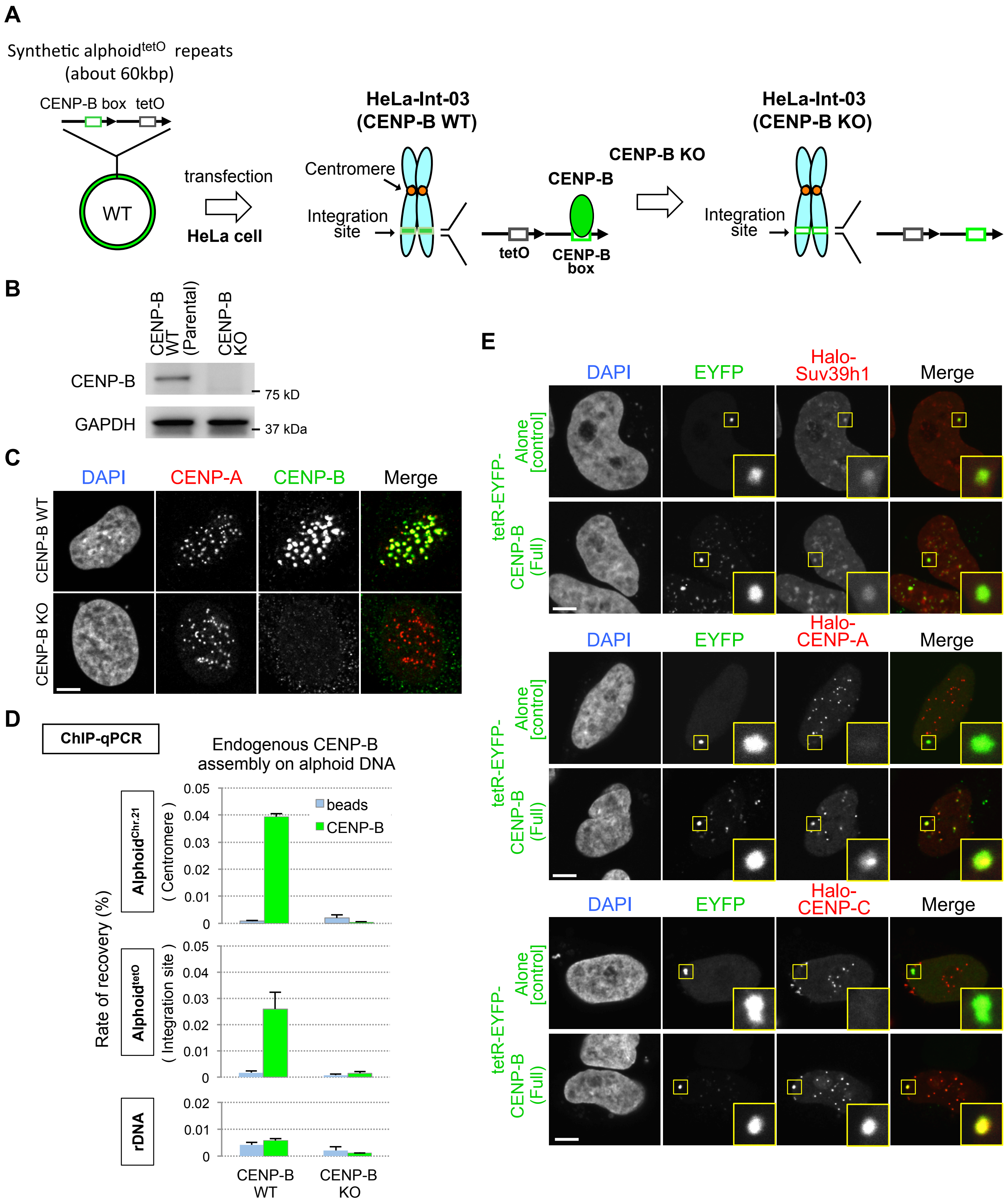

Journal of Cell Science • Supplementary information

Figure S1

Effectiveness evaluation of FMIT assay on the screening proteins for assembling on ectopic alphoid^{tetO} DNA in a CENP-B-dependent manner.

(A) Schematic drawing of strain construction. HeLa-Int-03 has an ectopic integration site of alphoid^{tetO} DNA repeats. This cell line was established in the previous study (Ohzeki et al., 2012). CENP-B gene was knocked out by CRISPR/Cas9 system. CENP-B WT and KO indicate CENP-B wild type and knockout, respectively. (B, C and D) Knockout of CENP-B gene in HeLa-Int-03 was confirmed by immunoblotting (B) and Immunostaining (C) and ChIP-qPCR assay (D) using antibodies against indicating proteins. (C) Scale bar, 5μm. (D) Recovered DNAs were quantified by real-time PCR using primer set for alphoid^{tetO}, alphoid$^{chr.21}$ and rDNA (ribosomal DNA). Rate of recovery represents IP/input (%). Results are mean ± S.E.M. (*n*=3 experiments). (E) Representative images of tetR-EYFP-CENP-B-dependent assembly of Halo-Suv39h1, -CENP-A and -CENP-C on alphoid^{tetO} in FMIT assay using HeLa-Int-03 CENP-B KO strain. The yellow square indicates the tetR-EYFP-protein spots (green) on alphoid^{tetO} DNA site. Scale bars, 5μm.

Journal of Cell Science • Supplementary information

Figure S2

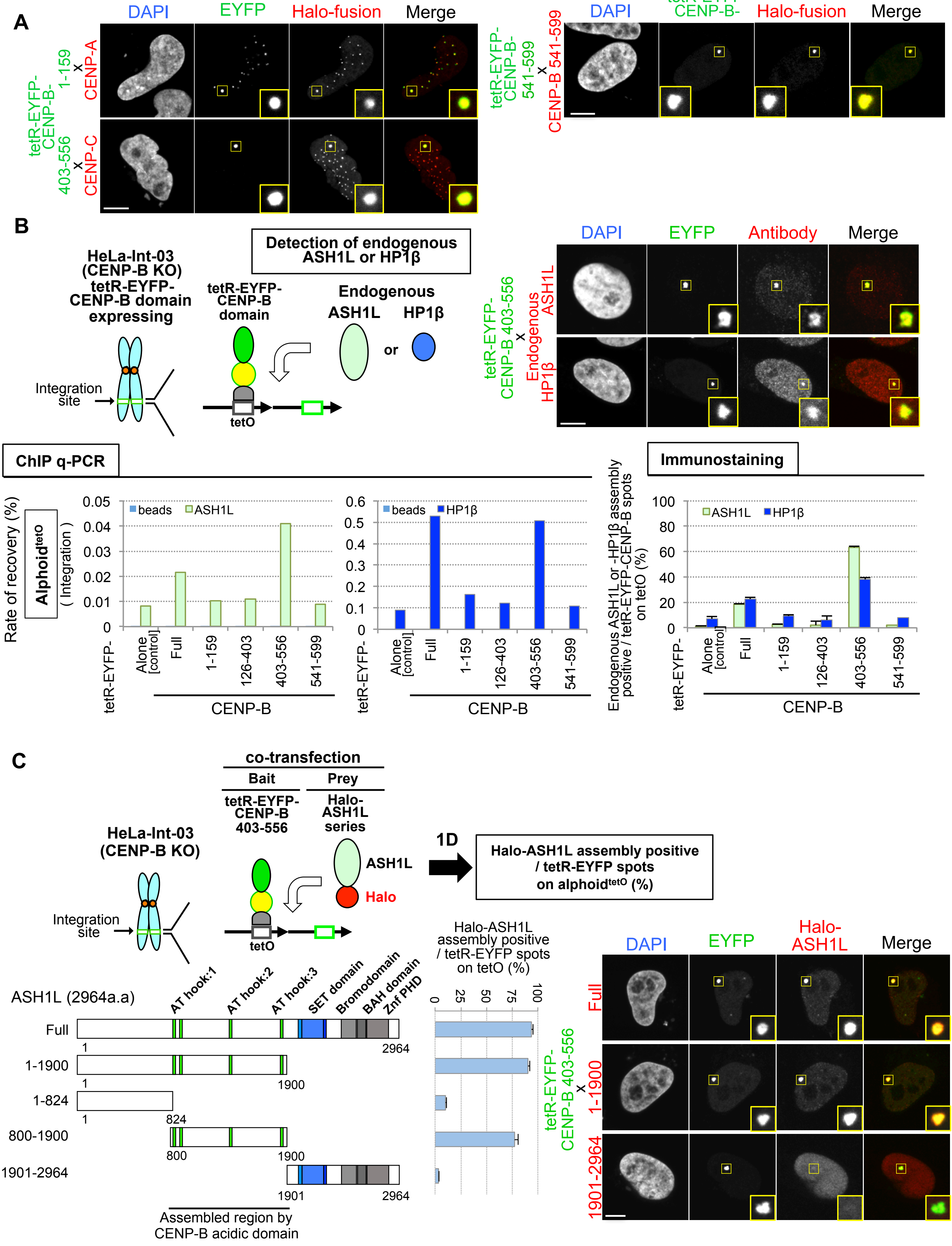

Journal of Cell Science • Supplementary information

Figure S2

FMIT assay related to Figure 3.

(A) Representative images of co-transfected cell with expression plasmid of tetR-EYFP-CENP-$B^{1\text{-}159}$ and Halo-CENP-A (upper left), tetR-EYFP-CENP-$B^{403\text{-}556}$ and Halo-CENP-C (lower left), or tetR-EYFP-CENP-$B^{541\text{-}599}$ and Halo-CENP-$B^{541\text{-}599}$ (right) in FMIT assay (Fig.3 C). Cells were stained with DAPI and Halo-TMR ligand. The yellow square indicates the tetR-EYFP-protein spots (green) on $\text{alphoid}^{\text{tetO}}$ DNA integration site. Scale bars, 5μm. (B) Verification of CENP-B dependent assembly of endogenous ASH1L and HP1β. HeLa-Int-03 CENP-B KO cells expressing tetR-EYFP-Alone or -CENP-B domain series (upper left) were analyzed by cytology (upper right panel and lower right panel) and ChIP-qPCR assay (*n*=1 experiment) (lower left panel) using antibodies against ASH1L or HP1β. For the cytology; The cells were stained with DAPI, anti-ASH1L or - HP1β antibody (red). The yellow square indicates the tetR-EYFP-CENP-B spots (green) on $\text{alphoid}^{\text{tetO}}$ DNA integration site. Scale bars, 5μm. More than 100 cells were counted to obtain the frequency of assembly for each assay (lower right panel). Results are mean ± S.E.M. (*n*=3 experiments). (C) Identification of ASH1L region assembled by CENP-B acidic domain tethering. Schematic drawing of FMIT assay (upper) and tested ASH1L protein domains (lower left). Lower middle; Frequency of Halo-ASH1L assembly on $\text{alphoid}^{\text{tetO}}$ integration site at 24 hours after transfection. More than 50 cells were counted for each assay. Results are mean ± S.E.M. (*n*=3 experiments). Lower right; Representative images of co-transfected cell of tetR-EYFP-CENP-$B^{403\text{-}556}$ expression plasmid and Halo-ASH1L series expression plasmid. The yellow square indicates the tetR-EYFP-protein spots (green) on $\text{alphoid}^{\text{tetO}}$ DNA integration site. Scale bars, 5μm.

Journal of Cell Science • Supplementary information

Figure S3

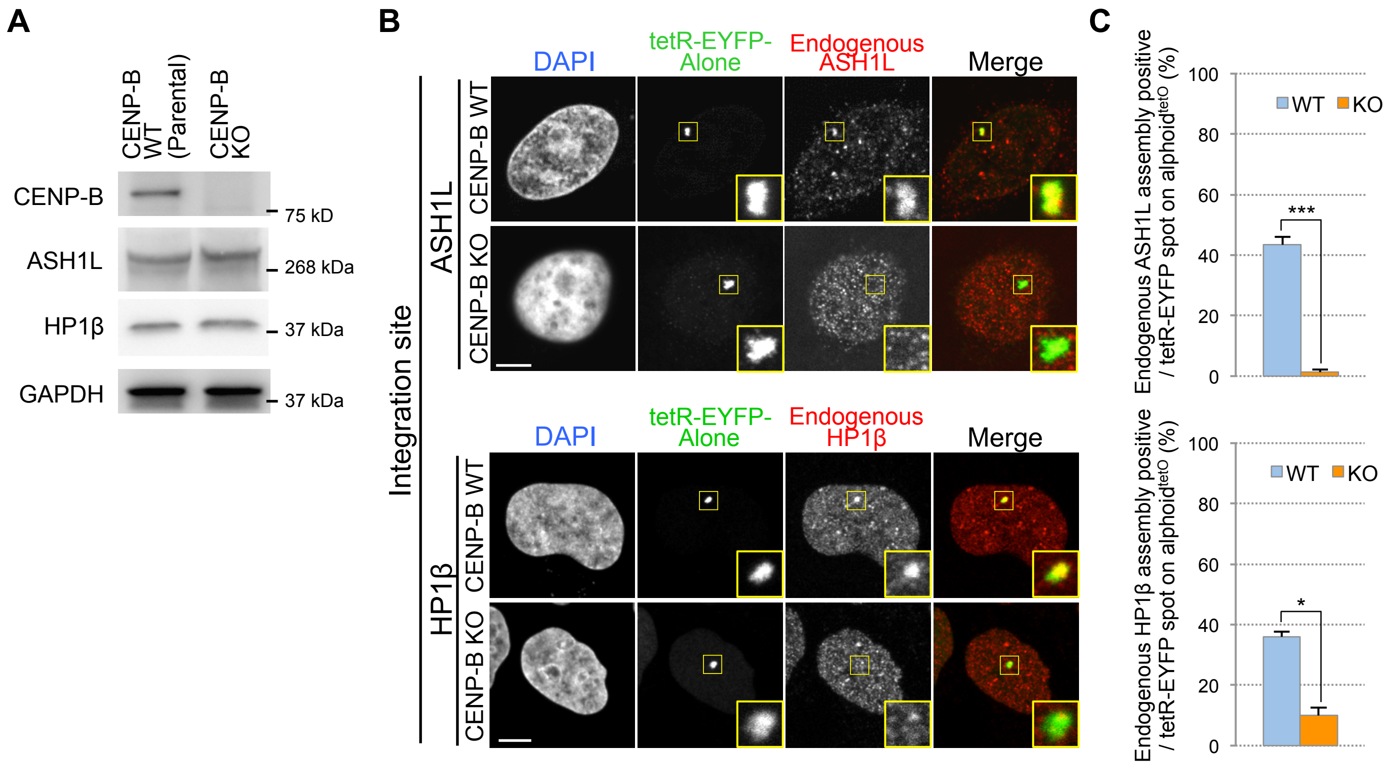

Figure S3

CENP-B dependent localization of ASH1L and HP1 on ectopic alphoid^{tetO} DNA integration site.

(A) Immunoblot analyses of ASH1L and HP1β in CENP- B WT and CENP-B KO cell line. The antibodies used are indicated on the left. No significant difference was detected in total protein levels of ASH1L and HP1β between CENP-B WT and KO cell lines. (B and C) Detection of endogenous ASH1L or HP1β at ectopic alphoid^{tetO} DNA integration site. tetR-EYFP-Alone expressing plasmids were transfected into HeLa-Int-03 cell line of CENP-B WT or KO. (B) The cells were stained with DAPI and anti-ASH1L or HP1β antibody (red). Yellow squares indicate the tetR-EYFP-protein spots (green) on alphoid^{tetO} DNA site. Scale bars, 5 µm. (C) Frequency of endogenous ASH1L or HP1β assembly on the ectopic alphoid^{tetO} DNA integration site. Endogenous signals on EYFP-spot of over 50 cells were counted. Results are mean ± S.E.M. (*n*=3 experiments). P-values (*t*-test, two-tailed) are indicated by asterisk. *$p < 0.05$, ***$p < 0.005$.

Journal of Cell Science • Supplementary information

Figure S4

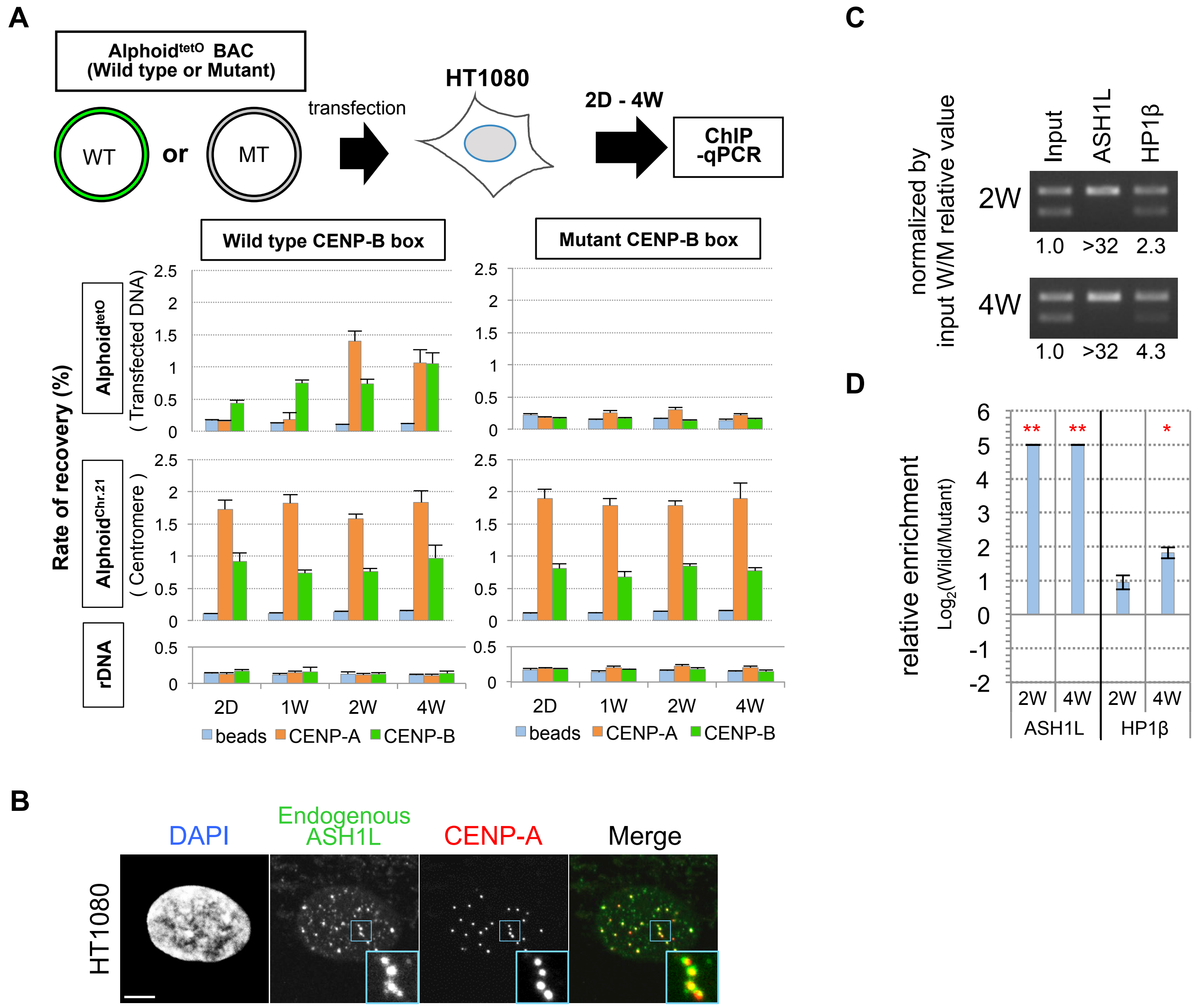

Figure S4

ChIP and cytology related to Figure 7.

(A) CENP-B dependent de novo CENP-A assembly on introduced alphoid^{tetO} DNA in HT1080. Upper panel; Schematic drawing of this assay. Wild type or mutant CENP-B box alphoid^{tetO} DNA was individually transfected into HT1080 cells. Lower panel; ChIP analyses were carried out with anti-CENP-A antibody (CENP-A), anti-CENP-B antibody (CENP-B) or without antibody (beads). Results are mean ± S.E.M. (*n*=3 experiments). (B) Detection of endogenous ASH1L at centromere in HT1080 cells. The cells were stained with anti-ASH1L (green) and anti-CENP-A antibodies (red) as same as Figure 4 A. Scale bar, 5μm. (C and D) ASH1L and HP1β assemble on the introduced alphoid^{tetO} DNA depending on CENP-B binding. ASH1L and HP1β were analyzed by ChIP and competitive PCR described in Fig. 7. After 2 weeks (2W) and 4 weeks (4W) of transfection, cells

Journal of Cell Science • Supplementary information

were analyzed with anti-ASH1L or -HP1β antibody. Results are mean ± S.E.M. (*n*=3 experiments). P-values (*t*-test, two-tailed) are indicated by asterisk. *p< 0.05, **p< 0.01. Red asterisk indicates 0< WT/MT log_2 ratio.

Figure S5

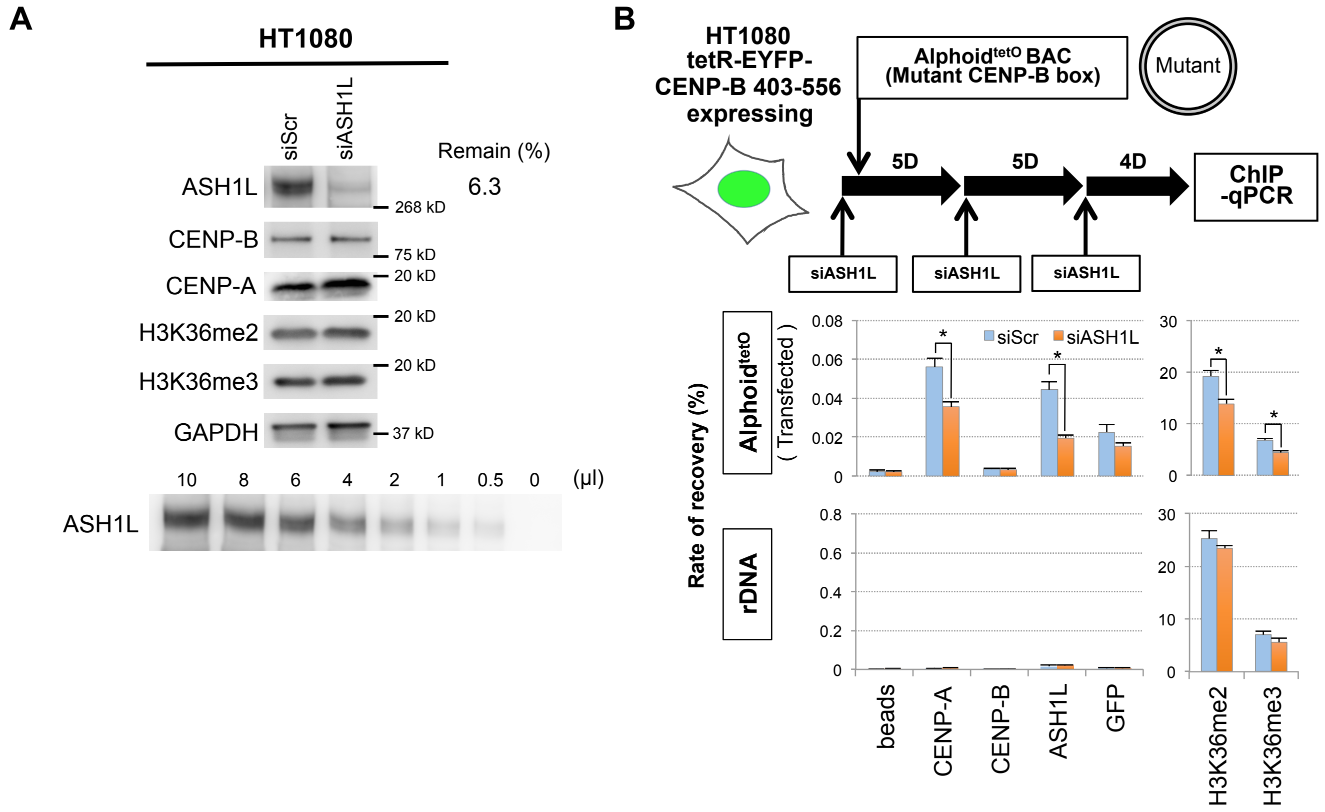

Figure S5

De novo CENP-A assembly on introduced alphoid DNA decreased by ASH1L-knockdown. (A) Effect of ASH1L depletion by siRNA on protein expression level. The HT1080 cells were harvested at 2 weeks after the first transfection of siRNA. Each protein level was detected by western blotting using the antibody indicated in the left. ASH1L-knockdown level was calculated from the dilution series of whole cell extract. GAPDH, internal control. (B) De novo CENP-A assembly was affected by ASH1L-knockdown. Quantification of de novo CENP-A assembly on introduced mutant CENP-B box alphoid^{tetO} DNA by ChIP assay. ChIP analyses were carried out with the antibody indicated below or without antibody (beads). Recovered DNAs were quantified by real-time PCR using primer set for alphoid^{tetO} and rDNA. Results are mean ± S.E.M. (n=3 experiments). P-values (t-test, two-tailed) are indicated by asterisk. *$p < 0.05$.

Journal of Cell Science • Supplementary information
