# Supplementary material for: CENP-B creates alternative epigenetic chromatin states permissive for CENP-A or heterochromatin assembly
Source: J Cell Sci. 2020 Aug 11;133(15):jcs243303. doi: 10.1242/jcs.243303 (PMC7438015; doi:10.1242/jcs.243303)
Supplement: Reviewer comments [file joces-133-243303_review_history.pdf]

CENP-B creates alternative epigenetic chromatin states permissive for CENP-A or heterochromatin assembly

Koichiro Otake, Jun-ichiro Ohzeki, Nobuaki Shono, Kazuto Kugou, Koei Okazaki, Takahiro Nagase, Hisashi Yamakawa, Natalay Kouprina, Vladimir Larionov, Hiroshi Kimura, William C Earnshaw and Hiroshi Masumoto
DOI: 10.1242/jcs.243303

Editor: David Glover

Review timeline
Original submission: 23 December 2019
Editorial decision: 27 January 2020
First revision received: 26 May 2020
Accepted: 29 June 2020

Original submission

First decision letter

MS ID#: JOCES/2019/243303

MS TITLE: CENP-B creates a bistable chromatin state permissive for CENP-A or heterochromatin assembly

AUTHORS: Koichiro Otake, Jun-ichiro Ohzeki, Nobuaki Shono, Kazuto Kugou, Koei Okazaki, Takahiro Nagase, Hisashi Yamakawa, Natalay Kouprina, Vladimir Larionov, Hiroshi Kimura, William C Earnshaw, and Hiroshi Masumoto
ARTICLE TYPE: Research Article

We have now reached a decision on the above manuscript.

To see the reviewers' reports and a copy of this decision letter, please go to: https://submit-jcs.biologists.org and click on the 'Manuscripts with Decisions' queue in the Author Area. (Corresponding author only has access to reviews.)

As you will see, the reviewers raise a number of substantial criticisms that prevent me from accepting the paper at this stage. They suggest, however, that a revised version might prove acceptable, if you can address their concerns. If you think that you can deal satisfactorily with the criticisms on revision, I would be pleased to see a revised manuscript. We would then return it to the reviewers.

Please ensure that you clearly highlight all changes made in the revised manuscript. Please avoid using 'Tracked changes' in Word files as these are lost in PDF conversion.

I should be grateful if you would also provide a point-by-point response detailing how you have dealt with the points raised by the reviewers in the 'Response to Reviewers' box. Please attend to all of the reviewers' comments. If you do not agree with any of their criticisms or suggestions please explain clearly why this is so.

© 2020. Published by The Company of Biologists under the terms of the Creative Commons Attribution License (https://creativecommons.org/licenses/by/4.0/).

Reviewer 1

Advance summary and potential significance to field

CENP-B is a sequence specific DNA binding protein and recognizes a 17bp motif of alphoid DNA in human centromere region. CENP-B also binds to CENP-A and CENP-C, and CENP-B appears to contribute to kinetochore formation. However, CENP-B knockout (KO) mice are viable and chromosome segregation occurs in CENP-B KO mice, suggesting that CENP-B is dispensable for maintenance of kinetochore function. While these data were paradoxical, authors' group and others showed that CENP-B contributes to de novo centromere formation using a human artificial chromosome (HAC). On the other hand, CENP-B also recruits Suv39 (histone methyl transferase) to centromeres, which induces heterochromatin and invades kinetochore function. Based on these results, CENP-B appears to have two opposite roles (facilitating centromere formation and suppressing centromere function via hetrochromatinaization). However, it is still unclear how CENP-B controls such "bistable" chromatin states.

In this study, authors found that the CENP-B acidic domain binds ASH1L, which is H3K36 methylase, as well as Suv39h1 and HP1. H3K36 methylation is detected in open chromatin regions, while H3K9 methylation and HP1 are detected in closed heterochromatin regions. When authors tethered ASHL1 to a particular locus on the HAC, H3K9 methylation levels decreased. By contrast, they detected low levels of H3K36 methylation at an H3K9 methylation-rich locus on the HAC. Authors proposed a molecular model by which CENP-B balances CENP-A assembly and heterochromatin formation, which might be important de novo centromere formation.

This paper contains a new finding that the CENP-B acidic domain recruits ASH1L, which is interesting. I think that this new finding is worth to publish in JCS. However, it is still hard to understand how CENP-B controls "bistable" chromatin states. Authors should give comments on this issue in the revised version.

Comments for the author

CENP-B is a sequence specific DNA binding protein and recognizes a 17bp motif of alphoid DNA in human centromere region. CENP-B also binds to CENP-A and CENP-C, and CENP-B appears to contribute to kinetochore formation. However, CENP-B knockout (KO) mice are viable and chromosome segregation occurs in CENP-B KO mice, suggesting that CENP-B is dispensable for maintenance of kinetochore function. While these data were paradoxical, authors' group and others showed that CENP-B contributes to de novo centromere formation using a human artificial chromosome (HAC). On the other hand, CENP-B also recruits Suv39 (histone methyl transferase) to centromeres, which induces heterochromatin and invades kinetochore function. Based on these results, CENP-B appears to have two opposite roles (facilitating centromere formation and suppressing centromere function via hetrochromatinaization). However, it is still unclear how CENP-B controls such "bistable" chromatin states.

In this study, authors found that the CENP-B acidic domain binds ASH1L, which is H3K36 methylase, as well as Suv39h1 and HP1. H3K36 methylation is detected in open chromatin regions, while H3K9 methylation and HP1 are detected in closed heterochromatin regions. When authors tethered ASHL1 to a particular locus on the HAC, H3K9 methylation levels decreased. By contrast, they detected low levels of H3K36 methylation at an H3K9 methylation-rich locus on the HAC. Authors proposed a molecular model by which CENP-B balances CENP-A assembly and heterochromatin formation, which might be important de novo centromere formation.

This paper contains a new finding that the CENP-B acidic domain recruits ASH1L, which is interesting. I think that this new finding is worth to publish in JCS. However, it is still hard to understand how CENP-B controls "bistable" chromatin states. Authors should give comments on this issue in the revised version. My specific comments are:

1. From Figure 1 to 3, authors performed elegant works, and identified ASH1L. Figure 4 experiments are also interesting. It is clear that both ASH1L and HP1 localize endogenous centromeres in CENP-B dependent manner. However, I am curious how ASH1L and HP1 localize at

© 2020. Published by The Company of Biologists under the terms of the Creative Commons Attribution License (https://creativecommons.org/licenses/by/4.0/).

same centromeres. If authors can co-stain ASH1L and HP1 in each cell, authors would be able to address this question.
2. Authors concluded that ASH1L(800-1900) is good binding site for CENP-B (Figure S3). But, ASH1L (1901-2961) was used in following Figure 5 experiments. It is slightly odd and results may be changed if they use ASH1L(800-1900).
3. Figure 7 experiments are elegant. But, again I am curious about a balance between ASH1L and HP1 after 2W. H3K36me2/3 is dominant on WT CENP-B box, suggesting that ASH1L might be dominant to HP1 on this region. Can authors show such a data?
4. In Final HAC formation experiments, author concluded that both the N-terminal and acid domain are required for HAC formation. But, it may be hard to say such a conclusion, because analyzed cell numbers are too small. If they analyzed more cells, the acidic domain may induce HAC formation in some of cells. Therefore, they should carefully revise their conclusion.

Reviewer 2

Advance summary and potential significance to field

In most eukaryotes, centromere identity is maintained by epigenetic mechanisms (i.e. DNA-sequence independent mechanisms). The main epigenetic marker is CENP-A, a histone H3 variant which, through contacts with various proteins, can seed the assembly of the kinetochore. The dissection of the mechanisms of CENP-A deposition required to maintain the centromere epigenetic marker, and how such mechanisms are influenced by chromatin status, is important and timely. Although usually centromeres are not established de novo, several studies have focused on de novo establishment as a means to address the chromatin requirements for centromere formation. The work described in this manuscript belongs to this branch of studies. Otake and co-workers report a large body of observations on the role of CENP-B in centromere establishment. CENP-B is the only sequence-specific DNA-binding protein in the centromere and while not required for centromere maintenance, it has been previously implicated in centromere establishment. Building on assay already used in their previous studies, the authors conclude that CENP-B has a role in the recruitment of various chromatin modifiers and readers that influence CENP-A deposition.
The authors focused in particular on ASH1L (H3K36 methylase) and HP1 (H3K9me binder). The CENP-B acidic domain emerged for its crucial role in these interactions

Collectively, this study is thorough and a technical tour-de-force. The topic is complex and the tools available to dissect it limited. In this respect, the authors' effort is commendable and quite impressive, and several of the conclusions are interesting. On the short side, there is no strong mechanistic result. The role of the CENP-B acidic domain seems proven, but how it works remains unclear. I also feel that the insistence on "bistability" is misplaced and that the authors could improve the narration to make the manuscript more accessible. As written, it is a quite hard read.

Comments for the author

-Title, abstract, and main body I am not sure that "bistable" is the appropriate word to define the effects of CENP-B. The reason why "bistable" feels awkward is that the authors seem to imply that the right balance of two opposing activities (ASHL and HP1) may be required for the stability of centromeres. Both activities are required, while if one prevails, centromeres are destabilized. It is difficult to see any bistability (the existence of two stable steady states) in this system. It also does not help that the authors use "and/or" in the (last line of the) abstract.

-Introduction, page 4
"However, CENP-C also binds to the carboxyl tail nucleosomal CENP-A…Thus, the CENP-C/CENP-A direct interaction can bypass the necessity for CENP-B" This is correct of course, but there are additional contacts of CCAN subunits with CENP-A, most notably CENP-N, that are also likely to play a role, and the authors should mention this body of knowledge.

-Introduction, page 5
I am unclear about the "conflicting chromatin states" referred to by the authors. The authors refer to experiments (Nakano et al. 2008 and other cited references) in which tethering heterochromatin factors to a tetO locus within an alphoid sequence was shown to inhibit CENP-A incorporation. They

© 2020. Published by The Company of Biologists under the terms of the Creative Commons Attribution License (https://creativecommons.org/licenses/by/4.0/).

also refer to other experiments (Okada et al. 2007) in which re-expression of CENP-B in cells lacking CENP-B caused increased deposition of H3K9me3. The authors' argument, if I am correct, is that CENP-B can also promote heterochromatinization that counteracts CENP-A deposition. As a reminder, however, Okada et al. 2008 showed that the re-expression of CENP-B promoted formation of CENP-A chromatin. Thus concomitant deposition of H3K9me3 must have been compatible with CENP-A deposition in this case. It seems to me that the comparison of these two very different types of experiments does not lead to a very clear initial hypothesis.

-Figure 1A FMIT assay: I expect TetR-EYFP-CENP-B will not only target the tetO site but also the CENP-B box (or boxes?) at the ectopic site, but this is not shown in the schematic. Also, kinetochore levels of the TetR-EYFP-CENP-B construct seems to vary considerably in the two IFs. Does this reflect differences in expression levels in different cells?

-Page 6, middle section The authors could elect to explain that these experiments were done in the presence of CENP-B, instead of letting the reader figure this out by examining Figure 2A. In addition, the latter is confusing enough:
what is the green oval? CENP-B? In this case, please label it. What are the arrows meant to represent?

-Figure 2 Here the authors resort to CENP-B-positive cells, while one may argue, based on their model, that a requirement for CENP-B ought to be bypassed if ASH1L or HP1 were recruited artificially through the TetO site. Have these experiments been carried out also in cells lacking CENP-B?

-Figure 3 Here the authors return to the FMIT assay in CENP-B knockout cells presented in Figure 1. I wonder if this should not come as Figure 2 instead. As in Figure 1, here the authors are comparing the recruitment of candidate Halo-tagged proteins to the TetO site when various CENP-B segments are targeted there and implicate the acidic domain in the recruitment of ASH1L and HP1. They neglect potential effects from binding of CENP-B to the CENP-B box(es) in the neighboring alphoid DNA at the ectopic site in this process (full length CENP-B and the acidic domain may not work through the same mechanism if binding to alphoid DNA contributes to the observed effects).

-Figure 4 Here the authors show an interesting new result, namely CENP-B dependent recruitment of ASH1L to centromeres. This is a bit anecdotal, though, because the authors do not investigate the cell cycle dependence of the recruitment and there is no quantification of the results for a comparable set of conditions in the CENP-B positive and the CENP-B negative cell lines. The same is true for the HP1-beta recruitment.

The ChIP experiments should be marked as different panels. These experiments come without associated statistics.

-Figure 5 In panel B, but not in A (nor in previous figures depicting experiments in CENP-B expressing cells), the green oval that I assume could correspond to CENP-B now appears on the alphoid array. In the corresponding IFs, neither ASH1L nor HP1beta appear to localize to centromeres any longer, while they appeared to do so in Figure 4.

Minor points
-Page 6
“A control dimer domain” should read “a control dimerization domain”
“…histone chaperons (Mis18bp1…” Technically Mis18BP1 is not a histone chaperone but a recruitment factor for a histone chaperone

© 2020. Published by The Company of Biologists under the terms of the Creative Commons Attribution License (https://creativecommons.org/licenses/by/4.0/).

First revision

Author response to reviewers' comments

Dear Dr. Glover,

Thank you very much for giving us this opportunity to revise our MS.
We have done our best to respond the reviewers' valuable comments and believe that the revised manuscript has been greatly improved by rewriting and adding new data (new Fig 4B,D,E,F,G and Fig S4C,D). A point-by-point response is included with reviewers' comments. We thank you and all the reviewers for your time evaluating and helping to improve our MS.

We look forward to receiving a hopefully positive decision from you when you have had time to consider our revised MS.

Best wishes, Hiroshi
Masumoto

Reviewer 1 Advance Summary and Potential Significance to Field:
CENP-B is a sequence specific DNA binding protein and recognizes a 17bp motif of alphoid DNA in human centromere region. CENP-B also binds to CENP-A and CENP-C, and CENP-B appears to contribute to kinetochore formation. However, CENP-B knockout (KO) mice are viable and chromosome segregation occurs in CENP-B KO mice, suggesting that CENP-B is dispensable for maintenance of kinetochore function. While these data were paradoxical, authors' group and others showed that CENP-B contributes to de novo centromere formation using a human artificial chromosome (HAC). On the other hand, CENP-B also recruits Suv39 (histone methyl transferase) to centromeres, which induces heterochromatin and invades kinetochore function. Based on these results, CENP-B appears to have two opposite roles (facilitating centromere formation and suppressing centromere function via hetrochromatinaization). However, it is still unclear how CENP-B controls such "bistable" chromatin states.

In this study, authors found that the CENP-B acidic domain binds ASH1L, which is H3K36 methylase, as well as Suv39h1 and HP1. H3K36 methylation is detected in open chromatin regions, while H3K9 methylation and HP1 are detected in closed heterochromatin regions. When authors tethered ASHL1 to a particular locus on the HAC, H3K9 methylation levels decreased. By contrast, they detected low levels of H3K36 methylation at an H3K9 methylation-rich locus on the HAC. Authors proposed a molecular model by which CENP-B balances CENP-A assembly and heterochromatin formation, which might be important de novo centromere formation.

This paper contains a new finding that the CENP-B acidic domain recruits ASH1L, which is interesting. I think that this new finding is worth to publish in JCS. However, it is still hard to understand how CENP-B controls "bistable" chromatin states. Authors should give comments on this issue in the revised version.

My specific comments are:
1. From Figure 1 to 3, authors performed elegant works, and identified ASH1L. Figure 4 experiments are also interesting. It is clear that both ASH1L and HP1 localize endogenous centromeres in CENP-B dependent manner. However, I am curious how ASH1L and HP1 localize at same centromeres. If authors can co-stain ASH1L and HP1 in each cell, authors would be able to address this question.

We thank the referee for this important comment.
We did our best to respond all the comments during the limited period available to us.

Response:
We have improved our method for quantifying HP1β in the centromeric region. HP1β is distributed in a rather wider area of the centromere including the pericentromere, and does not localize as a clear focused spot like the CENPs and CCAN.

© 2020. Published by The Company of Biologists under the terms of the Creative Commons Attribution License (https://creativecommons.org/licenses/by/4.0/).

Using the new method shown in the New Fig. 4 A, the distribution of fluorescence intensity of ASH1L and HP1ß was quantified and integrated in a zone of radius 11 pixels from the centroid of each CENP-A signal for each of the 45 centromeric CENP-A signals of each cell. The New Fig.4 B shows that all CENP-B signals fall within this zone.

The New Fig.4 C and D shows the results of this quantitative analysis of ASH1L and HP1ß fluorescence intensity for 20 cells (CENP-B WT and CENP-B KO, respectively). The distributions of ASH1L and HP1ß fluorescence intensity were significantly higher close to the CENP-A signal in CENP-B WT cells. However, this significant difference was lost in CENP- B KO cells. This analysis also shows that ASH1L and HP1ß are not equally distributed around the centromeres of each cell.

Next, CENP-B, ASH1L and HP1ß were triple-stained and analyzed by the new method (New Fig. 4 E and F). This new study revealed that ASH1L fluorescence was weak in cells where HP1ß levels were higher near centromeres. Conversely, HP1ß fluorescence was weak in cells where ASH1L levels were higher near centromeres. These results suggest that ASH1L and HP1ß are distributed in a mutually exclusive manner at individual centromeres. These results fit better with the results of Figs 5 & 6.

We suspect that this mutually exclusive localization of ASH1L and HP1ß is explained by cell cycle differences. Currently, we are analyzing such cell cycle differences, but it will take more time to produce the results under the present limited working conditions.

We thank the referee for asking this question, which has enabled us to better integrate the results of our experiments.

2. Authors concluded that ASH1L(800-1900) is good binding site for CENP-B (Figure S3). But, ASH1L (1901-2961) was used in following Figure 5 experiments. It is slightly odd and results may be changed if they use ASH1L(800-1900).

Response:
The main purpose of this manuscript is to search for and to identify factors involved in two different chromatin states produced by CENP-B binding, which has been poorly studied so far. To do this, we tethered CENP-B to an ectopic tetO integration site and identified factors recruited by it. We identified ASH1L and HP1 as such candidates.

In Fig. 2, we showed that tethering ASH1L 1901-2964 increased CENP-A at the HAC centromere, but ASH1L 1-1900 (including 800-1900) did not (Fig.2 C and D). An interaction domain of ASH1L with CENP-B was identified in aa 800-1900 (Fig. S2 C). ASH1L is a large protein and, unfortunately, we failed to express full-size ASH1L containing 800-1900 efficiently in the cells.

However, in experiments performed during revision, we found that endogenous ASH1L and HP1ß are distributed at the centromeres in a mutually exclusive manner, as shown in the new Fig.4 E and F. This confirms results obtained by tethering ASH1L 1901-2964 (without aa 800-1900) tethering to the integration site (Fig. 5 B, C, D). We show in Fig. 5A that H3K36 methylation by ASH1 and H3K9 methylation (which recruits HP1ß) also change conversely.

Moreover, when ASH1L is depleted by siRNA (Fig. 6 A), H3K36 methylation decreases and H3K9 methylation increases at centromeres - an effect opposite to that in Fig. 5A where ASH1L 1901-2964 was tethered to the integration site. Consistent with this, we have observed an assembly of ASH1L on CENP-B box-containing alphoid DNA accompanied by an increase in H3K36 methylation in the early stage of transfection (Fig.7 and new Fig.S4 C and D).

In summary, all the diverse results we have provided are consistent. These include the effect of ASH1L 1901-2964 tethering, our new observation of the mutually exclusive distribution of endogenous ASH1L and HP1ß, the opposite effects of ASH1L depletion and ASH1L 1901- 2964 tethering on H3K36me and H3K9me levels, and a CENP-B-dependent increase in ASH1L assembly and H3K36me levels.

We hope that we may be excused from delaying our MS by waiting to perform further speculative experiments using 800-1900.

© 2020. Published by The Company of Biologists under the terms of the Creative Commons Attribution License (https://creativecommons.org/licenses/by/4.0/).

3. Figure 7 experiments are elegant. But, again I am curious about a balance between ASH1L and HP1 after 2W. H3K36me2/3 is dominant on WT CENP-B box, suggesting that ASH1L might be dominant to HP1 on this region. Can authors show such a data?

Response:
We examined the CENP-B / CENP-B box-dependent assembly of ASH1L and HP1ß on the introduced alphoid DNA (New Fig. S4 C and D). Both ASH1L and HP1ß were preferentially assembled on alphoid DNA containing WT CENP-B boxes. This is in good agreement with the results of the FMIT assay shown in Figs. 1 & 3. ASH1L assembled more strongly than HP1ß on the alphoid DNA containing WT CENP-B boxes early after transfection (New Fig. S4 C and D). Thus, as pointed out by the reviewer, ASH1L appears to be dominant in the initial chromatin assembly on the introduced naked alphoid DNA in this HT1080 cell line.

4. In Final HAC formation experiments, author concluded that both the N-terminal and acid domain are required for HAC formation. But, it may be hard to say such a conclusion, because analyzed cell numbers are too small. If they analyzed more cells, the acidic domain may induce HAC formation in some of cells. Therefore, they should carefully revise their conclusion.

Response:
Further data on the HAC assay could not be carried out within this limited time frame. So, as the reviewer pointed out, we modified the text.

P. 13 middle: (revised MS, P. 14 top:)
Thus, tetR-fused full-size CENP-B tethered to mutant CENP-B box alphoid DNA via tetO retains the ability to assemble de novo CENP-A competent for functional centromere formation, but either the N-terminal DBD or the acidic domains alone **does this with reduced effectiveness. Further analysis is needed to determine if either domain alone has the ability to assemble functional centromere at low efficiency.**

P.14 middle: (revised MS, P. 15 top:)
We changed the text to read: **Importantly, full-length CENP-B containing both domains can efficiently induce functional centromere and HAC formation on transfected alphoid DNA.**

Reviewer 2 Advance Summary and Potential Significance to Field:
In most eukaryotes, centromere identity is maintained by epigenetic mechanisms (i.e. DNA-sequence independent mechanisms). The main epigenetic marker is CENP-A, a histone H3 variant which, through contacts with various proteins, can seed the assembly of the kinetochore. The dissection of the mechanisms of CENP-A deposition required to maintain the centromere epigenetic marker, and how such mechanisms are influenced by chromatin status, is important and timely. Although usually centromeres are not established de novo, several studies have focused on de novo establishment as a means to address the chromatin requirements for centromere formation. The work described in this manuscript belongs to this branch of studies. Otake and co-workers report a large body of observations on the role of CENP-B in centromere establishment. CENP-B is the only sequence-specific DNA- binding protein in the centromere, and while not required for centromere maintenance, it has been previously implicated in centromere establishment. Building on assay already used in their previous studies, the authors conclude that CENP-B has a role in the recruitment of various chromatin modifiers and readers that influence CENP-A deposition.
The authors focused in particular on ASH1L (H3K36 methylase) and HP1 (H3K9me binder). The CENP-B acidic domain emerged for its crucial role in these interactions
Collectively, this study is thorough and a technical tour-de-force. The topic is complex and the tools available to dissect it limited. In this respect, the authors' effort is commendable and quite impressive, and several of the conclusions are interesting. On the short side, there is no strong mechanistic result. The role of the CENP-B acidic domain seems proven, but how it works remains unclear. I also feel that the insistence on "bistability" is misplaced and that the authors could improve the narration to make the manuscript more accessible. As written, it is a quite hard read.

Reviewer 2 Comments for the Author:
-Title, abstract, and main body
I am not sure that "bistable" is the appropriate word to define the effects of CENP-B. The reason

© 2020. Published by The Company of Biologists under the terms of the Creative Commons Attribution License (https://creativecommons.org/licenses/by/4.0/).

why "bistable" feels awkward is that the authors seem to imply that the right balance of two opposing activities (ASHL and HP1) may be required for the stability of centromeres. Both activities are required, while if one prevails, centromeres are destabilized. It is difficult to see any bistability (the existence of two stable steady states) in this system. It also does not help that the authors use "and/or" in the (last line of the) abstract.

We thank the referee for this positive assessment of our study. In revision, we have worked hard to respond to the helpful and interesting points raised by the referees and also to make the MS easier to read.

Response:
We found in our present manuscript that CENP-B recruits various factors involved in the formation and maintenance of two different chromatin states, closed chromatin (heterochromatin) and open chromatin (permissive for CENP-A chromatin). Furthermore, as shown in new Fig.4 E and F, both ASH1L and HP1ß do not coexist at similar levels at centromeres of the same cell, but are distributed in a mutually exclusive manner depending on CENP-B binding. These results fit better with results of Figs 5 & 6. We had used the term "bistable" to describe this, but we understand that it could be difficult to understand precisely what we meant by using the term "bistable" in this context. So, we changed it to " **alternative epigenetic chromatin states**" or "**bidirectional chromatin**". This is easy to understand, as the reviewers pointed out.

Title was changed: CENP-B creates **alternative epigenetic** chromatin state**s** permissive for CENP-A or heterochromatin assembly

-Introduction, page 4
"However, CENP-C also binds to the carboxyl tail nucleosomal CENP-A…Thus, the CENP-C/CENP- A direct interaction can bypass the necessity for CENP-B" This is correct of course, but there are additional contacts of CCAN subunits with CENP-A, most notably CENP-N, that are also likely to play a role, and the authors should mention this body of knowledge.

Response:
In response to this comment by the reviewer, we added the following sentence:
CENP-A nucleosomes are stabilized by interactions with other CCAN components, including CENP-C (Carroll et al., 2010; Fachinetti et al., 2013) and CENP-N (Guo et al., 2017).

-Introduction, page 5
I am unclear about the "conflicting chromatin states" referred to by the authors. The authors refer to experiments (Nakano et al. 2008 and other cited references) in which tethering heterochromatin factors to a tetO locus within an alphoid sequence was shown to inhibit CENP-A incorporation. They also refer to other experiments (Okada et al. 2007) in which re-expression of CENP-B in cells lacking CENP-B caused increased deposition of H3K9me3. The authors' argument, if I am correct, is that CENP-B can also promote heterochromatinization that counteracts CENP-A deposition. As a reminder, however, Okada et al. 2008 showed that the re-expression of CENP-B promoted formation of CENP-A chromatin. Thus, concomitant deposition of H3K9me3 must have been compatible with CENP-A deposition in this case. It seems to me that the comparison of these two very different types of experiments does not lead to a very clear initial hypothesis.

Response:
In Okada et al. 2007, we showed that binding of CENP-B on transfected naked alphoid DNA causes strong de novo CENP-A assembly with de novo HAC formation. But binding of full- size CENP-B on ectopically integrated alphoid DNA suppresses de novo CENP-A assembly to a low level and induces strong H3K9me3 assembly. It is possible that the short-term effects of CENP-B involve interactions with dynamic protein partners, such as ASH1L, while the long term effects (especially on the ectopic integration site) involve establishment of a less dynamic repressive state (e.g. DNA methylation also promoting heterochromatin formation).

Furthermore, in HeLa cells, which have strong H3K9me3 methyltransferase activity, stronger H3K9me3 assembly occurred on the transfected alphoid DNA after 3 to 4 weeks, de novo CENP-A assembly was reversed and de novo HAC formation was suppressed. It was also shown that the tethering a HAT (antagonizing Suv39h1/H3K9methylase activity) on transfected alphoid DNA can

© 2020. Published by The Company of Biologists under the terms of the Creative Commons Attribution License (https://creativecommons.org/licenses/by/4.0/).

promote de novo CENP-A assembly and de novo HAC formation even in HeLa cells (Ohzeki et al 2012 EMBO J).

Thus, it is clear that CENP-A assembling conditions and strong heterochromatinization antagonize each other. However, in order to reduce confusion, the description of "conflicting chromatin state" has been changed to "It is unclear how CENP-B controls such different chromatin states."

-Figure 1A
FMIT assay: I expect TetR-EYFP-CENP-B will not only target the tetO site but also the CENP-B box (or boxes?) at the ectopic site, but this is not shown in the schematic. Also, kinetochore levels of the TetR-EYFP-CENP-B construct seems to vary considerably in the two IFs. Does this reflect differences in expression levels in different cells?

Response:
As the reviewer pointed out, we modified the figure to indicate that tetR-EYFP-CENP-B (Full) is also bound to the CENP-B box.

The FMIT assay can reproducibly show the same assembly characteristics despite differences in protein expression level. In this manuscript, we showed image stacks of 15 z- focal planes (covering a 5 µm depth) containing all CENP-A signals when observing the distribution of all endogenous centromeres of a cell. In contrast, when observing assembly to the integration site, we showed an image stack of five z-focal planes (covering a 1 µm depth) focused on the integration site in order to minimize overlap with the endogenous centromere signals.
As a result, the number of endogenous centromere signals in Fig.1A IF is different because the integration site and the endogenous centromeres do not always occupy the same focal plane. Thus, the number of endogenous centromere signals does not reflect differences in the expression level in different cells. Furthermore, there is unlikely to be a significant difference in the expression level of tetR-EYFP-CENP-B in different cells, as they show similar fluorescence levels at the integration site. Moreover, Fig. 3 also shows that both CENP-B (Full) and the acidic domain lacking the N-terminal DBD have a similar ability to recruit factors other than CENP-A. This supports the reliability of the FMIT assay for characterizing protein interactions on chromatin.

-Page 6, middle section
The authors could elect to explain that these experiments were done in the presence of CENP-B, instead of letting the reader figure this out by examining Figure 2A. In addition, the latter is confusing enough: what is the green oval? CENP-B? In this case, please label it. What are the arrows meant to represent?

Response:
We corrected the figure as pointed out by reviewer.
We labeled the CENP-B (green oval) and the meaning of the arrows. In addition, the figure was split into two to clarify whether tethering of an identified factor to the HAC increases or decreases endogenous CENP-A assembly levels.

-Figure 2
Here the authors resort to CENP-B-positive cells, while one may argue, based on their model, that a requirement for CENP-B ought to be bypassed if ASH1L or HP1 were recruited artificially through the TetO site. Have these experiments been carried out also in cells lacking CENP-B?

Response:

In the assay of Fig. 2, the tethering experiment was not performed on the HeLa HAC tetO-2- 4 strain where CENP-B was deleted. In Fig. 1, we obtained many candidate factors that assemble in a CENP-B-dependent manner at the ectopic integration site. The purpose of Fig.2 was to screen for factors that have a positive or negative effect on the centromeric CENP-A assembly levels promoted by our candidate factors. This objective was best achieved by observing the effects on CENP-A assembly at centromeres in CENP-B WT cells.

As a result, a new factor, ASH1L, that increases CENP-A and opposing factors, HP1s, that decrease CENP-A were identified as candidates for subsequent analyses. We do not believe that experiments

© 2020. Published by The Company of Biologists under the terms of the Creative Commons Attribution License (https://creativecommons.org/licenses/by/4.0/).

aimed at using the CENP-B KO to add back the functions lost from the centromere by tethering are necessary here. However, new data in Figure 4 also show how endogenous ASH1L and HP1 change distribution in CENP-B WTand KO cells.

-Figure 3
Here the authors return to the FMIT assay in CENP-B knockout cells presented in Figure 1. I wonder if this should not come as Figure 2 instead. As in Figure 1, here the authors are comparing the recruitment of candidate Halo-tagged proteins to the TetO site when various CENP-B segments are targeted there and implicate the acidic domain in the recruitment of ASH1L and HP1. They neglect potential effects from binding of CENP-B to the CENP-B box(es) in the neighboring alphoid DNA at the ectopic site in this process (full length CENP-B and the acidic domain may not work through the same mechanism if binding to alphoid DNA contributes to the observed effects).

Response:
Although would be possible to reorganize the manuscript as the reviewer suggests, as we answered in the previous comment, we obtained many candidate factors that assemble in a CENP-B-dependent manner at the ectopic integration site in Fig. 1. We chose to use Figure 2 to show why we selected ASH1L (a promoter of CENP-A assembly centromeres) and HP1 (a repressor of CENP-A assembly centromeres), for our subsequent analyses. Both organizations of the MS are possible, but we find our organization easier to understand.

In Fig. 3, tetR-EYFP-CENP-B divided into domains was expressed in CENP-B KO cells. CENP-B binds to the CENP-B box via the CENP-B DBD (1-159). The only constructs that can bind to the CENP-B box are CENP-B^{Full} and CENP-B^{1-159}. We confirmed that the other constructs were not assembled at the centromere when expressed in CENP-B KO cells. Therefore, for constructs other than CENP-B^{Full} and CENP-B^{1-159}, it was not necessary to consider effects due to binding adjacent CENP-B boxes.

In fact, the identified factors other than CENP-A (whose direct interaction with CENP-B^{1-159} was previously reported) showed almost the same assembly activity only with CENP-B^{Full} and CENP-$B^{acidic\ domain}$. Importantly, CENP-$B^{acidic\ domain}$ cannot bind to CENP-B boxes on its own. Furthermore, the CENP-B-dependent assembly activity of endogenous-ASH1L (and HP1ß) was also confirmed in Fig. 4 in both CENP-B WT and KO cells. Based on the above, it is clearly shown in Fig. 3 that binding of CENP-$B^{acidic\ domain}$ through tetO / tetR exerts the same activity as CENP-B^{Full} on the assembly of ASH1L and HP1ß.

Furthermore, in Fig.8, de novo CENP-A assembly activity was detected on mutant CENP-B box alphoid tetO when CENP-$B^{acidic\ domain}$ was tethered at a level similar to full length CENP-B. From this, we conclude that the CENP-$B^{acidic\ domain}$ alone exerts the same function as the acidic domain of CENP-B^{Full}. Thus, binding of CENP-B to CENP-B boxes is not required for the effects described here.

-Figure 4
Here the authors show an interesting new result, namely CENP-B dependent recruitment of ASH1L to centromeres. This is a bit anecdotal, though, because the authors do not investigate the cell cycle dependence of the recruitment and there is no quantification of the results for a comparable set of conditions in the CENP-B positive and the CENP-B negative cell lines. The same is true for the HP1- beta recruitment.
The ChIP experiments should be marked as different panels. These experiments come without associated statistics.

Response:
We thank the referee for this important comment.
We first improved the method for quantifying HP1ß near centromeres. HP1ß is distributed in a wider area of the centromere including the pericentromere, and does not localize as a clear spot like the CENPs or CCAN.

Therefore, we developed a new method shown in the New Fig. 4 A. The distribution of fluorescence intensity of ASH1L and HP1ß in a zone of radius 11 pixels surrounding the centroid of each CENP-A signal was quantified and integrated for each of the 45 centromeric CENP-A signals in each cell. The New Fig.4 B shows that all CENP-B signals fall within this zone.

© 2020. Published by The Company of Biologists under the terms of the Creative Commons Attribution License (https://creativecommons.org/licenses/by/4.0/).

The New Fig.4 C and D shows the results of this quantitative analysis of the ASH1L and HP1β fluorescence intensity for 20 cells (CENP-B WT and CENP-B KO, respectively). The distributions of ASH1L and HP1β fluorescence intensity were significantly higher close to the CENP-A signal in CENP-B WT cells. However, this significant difference was lost in CENP- B KO cells. This analysis also shows that ASH1L and HP1β are not equally distributed around the centromeres of each cell.

Next, CENP-B, ASH1L and HP1β were triple-stained and analyzed by the above method (New Fig. 4 E and F). This new study revealed that ASH1L fluorescence was weak in cells where HP1β levels were higher at centromeres, and conversely, HP1β fluorescence was weak in cells where ASH1L levels were higher at centromeres. These results suggest that ASH1L and HP1β are distributed around centromeres in a mutually exclusive manner. These results fit better with the results of Figs 5 & 6.

We suspect that this mutually exclusive localization of ASH1L and HP1β is caused by cell cycle differences, as the reviewers suggested. Currently, we are analyzing such cell cycle differences, but it would take more time to produce the results under the present limited working conditions.

Quantitative analysis by ChIP was performed 3 times in total, and statistical processing was performed and shown in different panels (Fig.4 G).

-Figure 5
In panel B, but not in A (nor in previous figures depicting experiments in CENP-B expressing cells), the green oval that I assume could correspond to CENP-B now appears on the alphoid array. In the corresponding IFs, neither ASH1L nor HP1beta appear to localize to centromeres any longer, while they appeared to do so in Figure 4.

Response:
The cells in Fig. 5A are using CENP-B WT. We have now labeled CENP-B in the diagram.

In IF in Fig. 5C, the centromere signals of ASH1L and HP1β are less clear than in Fig. 4 C because the focus was set on the integration site. This is the same reason as the response to the point in Fig. 1A observing the centromere signal of the whole cell.
In Fig.4 C, a stack of 15 z-focal plane images (a depth of 5 μm) was used to observe all centromere signals for the whole cell. The original Fig.4 A (lower) and B (lower) (New Fig.S3 B) and Fig.5 C are focused on the ectopic integration site. There, an image stack of five focal plane images (a depth of 1 μm) focusing on the integration site is shown in order to minimize overlap with the internal centromere signals. As a result, other centromere areas are not necessarily included in this image.

Minor points
-Page 6
"A control dimer domain" should read "a control dimerization domain" "...histone chaperons (Mis18bp1..." Technically Mis18BP1 is not a histone chaperone but a recruitment factor for a histone chaperone

Response:
We corrected these points as pointed out by the reviewer.

Second decision letter

MS ID#: JOCES/2019/243303

MS TITLE: CENP-B creates alternative epigenetic chromatin states permissive for CENP-A or heterochromatin assembly

© 2020. Published by The Company of Biologists under the terms of the Creative Commons Attribution License (https://creativecommons.org/licenses/by/4.0/).

AUTHORS: Koichiro Otake, Jun-ichiro Ohzeki, Nobuaki Shono, Kazuto Kugou, Koei Okazaki, Takahiro Nagase, Hisashi Yamakawa, Natalay Kouprina, Vladimir Larionov, Hiroshi Kimura, William C Earnshaw, and Hiroshi Masumoto
ARTICLE TYPE: Research Article

I am happy to tell you that your manuscript has been accepted for publication in Journal of Cell Science, pending standard ethics checks.

Reviewer 1

Advance summary and potential significance to field

Otake et al. substantially revised a previous MS with some key additional experiments. I feel that the MS is improved well. I really love new Figure 4. In addition, words “alternative epigenetic chromatin” are better than before.

Comments for the author

I recommend the current MS for publication in JCS.

Reviewer 2

Advance summary and potential significance to field

I am grateful to the authors for submitting an improved revised version of the manuscript and am happy to support publication of the manuscript. I congratulate the authors for their effort.

Comments for the author

No comments needed

© 2020. Published by The Company of Biologists under the terms of the Creative Commons Attribution License (https://creativecommons.org/licenses/by/4.0/).
